# Early detection and recovery of river herring spawning habitat use in response to a mainstem dam removal

**DOI:** 10.1371/journal.pone.0284561

**Published:** 2023-05-03

**Authors:** Claire S. Huang, Henry D. Legett, Louis V. Plough, Rob Aguilar, Catherine Fitzgerald, Benjamin Gregory, Keira Heggie, Benjamin Lee, Kimberly D. Richie, William Harbold, Matthew B. Ogburn

**Affiliations:** 1 Nicholas School of the Environment, Duke University Marine Lab, Beaufort, North Carolina, United States of America; 2 Smithsonian Environmental Research Center, Edgewater, Maryland, United States of America; 3 Horn Point Laboratory, University of Maryland Center for Environmental Science, Cambridge, Maryland, United States of America; 4 Maryland Department of Natural Resources, Annapolis, Maryland, United States of America; University of Hyogo, JAPAN

## Abstract

Historical loss of river and stream habitats due to impassable dams has contributed to the severe decline of many fish species. Anadromous fishes that migrate from the sea to freshwater streams to spawn have been especially impacted as dams restrict these fish from accessing ancestral spawning grounds. In 2018, Bloede Dam was removed from the Patapsco River near Baltimore, Maryland, restoring approximately 100 km of potential habitat for migratory fish. We assessed the response of anadromous river herring, alewife (*Alosa pseudoharengus*) and blueback herring (*Alosa aestivalis*), to this dam removal by monitoring environmental DNA (eDNA) and eggs from 2015 to 2021 at locations upstream and downstream of the dam site during their spawning migrations. We additionally assessed the presence of fish by collecting electrofishing samples and tracked the movements of individual adult fish within the river using passive integrated transponder (PIT) tags. No adult river herring, eDNA, or eggs were detected upstream of Bloede Dam in the four years prior to its removal despite the presence of a fish ladder. Our results suggest initial habitat use recovery by spawning river herring in the first year post-removal, although a relatively small proportion of the population in the river used the newly accessible habitat. In the three years post-removal, the likelihood of detecting river herring eDNA upstream of the former dam site increased to 5% for alewife and 13% for blueback herring. Two adult fish were also collected in electrofishing samples upstream of the dam site in 2021. We found no evidence of changes in egg abundance and no tagged fish were detected upstream of the dam site post-removal. While long term monitoring is needed to assess population changes, this study highlights the value of integrating methods for comprehensive understanding of habitat use following dam removal.

## Introduction

Connectivity between marine and river ecosystems is essential for the survival, growth, and productivity of anadromous fish species, which require migration access to multiple habitats to complete their life cycles [1]. However, the building of dams and other barriers has significantly fragmented habitats across watersheds and coastal areas—breaking the “river continuum” [2, 3]. Dams inhibit the upstream movement of migratory fishes and prevent access to freshwater spawning habitat and juvenile nursery grounds, resulting in significant loss of spawning biomass [4, 5]. In addition, historical loss of spawning habitat and productivity caused by dams further decreases fish population resilience to harvest mortalities and intensifying climate stressors [6].

Dam removal is a recognized—but challenging⁠—strategy to restore fish habitat connectivity and conserve anadromous species [7, 8]. Despite the construction of fish passage structures in dams, such as ladders and lifts, upstream passage efficiency remains at a low 20% for most anadromous species [9]. On the other hand, studies monitoring fish responses to dam removals have consistently observed that removing physical barriers restores longitudinal connectivity, allowing numerous migratory species to re-occupy upstream reaches [10–13]. A growing number of dam removal projects in North America have cited recovery of fish passage and ecological restoration as a primary goal [14], including Elwha Dam in Washington State, U.S. [13] and Edwards Dam in Maine [15].

Loss of habitat connectivity due to dams has long been identified as one of the primary threats to alewife (*Alosa pseudoharengus*) and blueback herring (*Alosa aestivalis*), collectively managed as river herring [8, 16–18]. These anadromous fish species are native to the Atlantic coast of North America and migrate every spring from the ocean to spawn in freshwater streams, lakes, and ponds [19]. Although river herring were once abundant throughout their range and supported one of the largest fisheries in the United States, fishery landings have decreased by over 90% in recent decades [20, 21]. The Chesapeake Bay region of the U.S. has experienced some of the most severe declines, where landings of river herring in 2009 were less than 1% of landings from 1950 to 1970, dropping from approximately 3.5 million to 45,000 lbs [21, 22]. While some populations in other regions, particularly New England stocks, have experienced slowly increasing trends in the past decade, river herring in the Chesapeake Bay remain at historical lows [21].

Due to consistently low abundances and their ecological and economic importance, river herring are a target for species recovery. So far, conservation efforts have largely focused on management changes through catch regulation. In 2005, the National Marine Fisheries Service (NMFS) declared alewife and blueback herring to be “Species of Concern,” and in 2012 a moratorium was imposed on the commercial and recreational fisheries in Maryland and Virginia [20]. Yet conservation interventions to reduce harvest mortality may not be sufficient in recovering river herring populations if the spawning population output remains low [5]. This is especially true for river herring, as forage species populations remain vulnerable to high natural mortality pressures as prey [5, 8, 23]. Modeled changes in New England alosine biomass show minimal responses to reduced fishing effort, whereas combining reduced fishing effort and substantial increases in freshwater-marine connectivity could increase biomass to early 1900’s baselines [24]. Dam removal thus likely plays an important role in restoring depleted river herring populations given the species’ ecology and life history.

Several previous studies have demonstrated that removing dams can restore spawning habitat for river herring. Adult alewife and blueback herring can return to newly accessible spawning habitat within two years of restoration [15, 25–28]. Successful river herring reproduction and juvenile nurseries have also been confirmed in upstream reaches of restored tributaries following dam removals [12, 25]. In addition, increases in river herring abundance have been documented at some rivers. For example, in the Kennebec River in Maine, river herring counts increased 228% in the five years following the removal of Edwards Dam in 1999 and 1425% in the ten years following the removal of Fort Halifax Dam in 2008, when combined with restocking efforts [15].

Given its success in other regions, river herring conservation in the Chesapeake Bay is now turning to dam removal. In 2014, five mid-Atlantic state governments and federal agency partners signed the Chesapeake Bay Watershed Agreement, committing to open 212 km (132 mi) of freshwater streams to fish passage every two years by 2025 [29]. There are no detailed studies, however, evaluating dam removal impacts on river herring spawning migration in any Chesapeake Bay watersheds. Because dam removals also face complex socio-economic, regulatory, and political hurdles, a better understanding of fish population responses using empirical data can inform environmental decision-making for restoration priorities [8, 14, 30].

In this study, we evaluate the response of alewife and blueback herring to the removal of Bloede Dam in the Patapsco River, Maryland. Prior to its removal, biological surveys conducted by the Maryland Department of Natural Resources (MD DNR) detected river herring below, but not above Bloede Dam [31]. Further, Denil-type fish ladders installed in the dam failed to facilitate river herring passage [32, 33]. Thus, the state of Maryland, federal agencies, and environmental nonprofits considered removing Bloede Dam as a critical step for the restoration of river herring and other anadromous fishes in the Patapsco River. We examine the spatial extent of river herring habitat use and spawning activity by applying four complimentary monitoring methods in a “before-after, downstream-upstream” study design spanning four years prior to the dam removal (2015–2018) and three years post-removal (2019–2021). As a secondary objective, we also assess whether alewife and blueback herring exhibit species-specific responses to the dam removal. Monitoring methods included: 1) environmental DNA (eDNA), 2) ichthyoplankton (fish eggs) collection, 3) boat electrofishing, and 4) passive integrated transponder (PIT) telemetry, which together provide information on habitat use from the population to individual level. Given the rapid use of restored habitat observed in other rivers [12, 13, 15], we similarly predict that river herring will re-occupy restored habitat in the Patapsco River within three years of the removal of Bloede Dam.

## Materials and methods

### Study area

**Fig 1:**
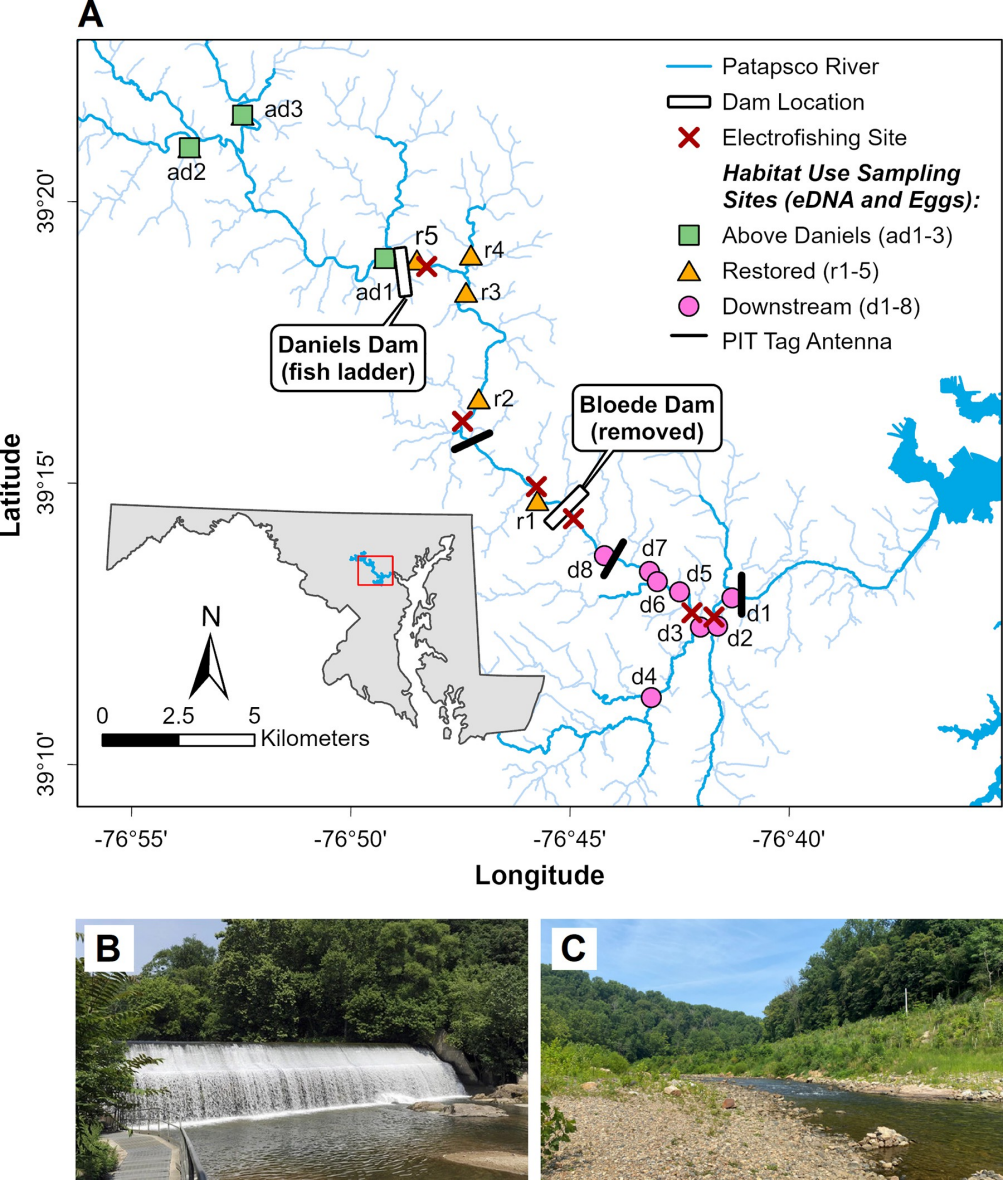
Map of general field sampling locations in the Patapsco River relative to the locations of Bloede Dam and Daniels Dam. The locations of sampling sites (A) were categorized into three groups according to their geographic location relative to the Bloede Dam site including “Downstream” of Bloede Dam, “Restored” sites between Bloede Dam and Daniels Dam, and “Above Daniels” sites upstream of Daniels Dam. Individual habitat use sampling sites for eDNA and ichthyoplankton are labeled by site code referenced in Table 1. Specific sites for electrofishing and PIT tag antennas can be found in S2 and S3 Figs. Images show Bloede Dam before removal in 2018 (B) and the former dam site after breaching and full bank and streambed restoration (C). Mapping layers (A) from Chesapeake Assessment and Scenario Tool (CAST) (2020), Maryland iMAP, Maryland Geological Survey, NOAA, Maryland Coastal Zone Management Program (2003), US Census Bureau (2018). Images (B, C) from Maryland Department of Natural Resources.

The Patapsco River, located in central Maryland, is a major tributary of the Chesapeake Bay and flows into Baltimore Harbor (Fig 1A). Historically, the Patapsco River valley was home to five dams (Bloede, Simkins, Union, Daniels, and Liberty) that serve as reservoirs or were used to power local flour and textile mills in the early 1900s. Starting in 2010, the Maryland Department of Natural Resources (DNR), American Rivers, Friends of the Patapsco Valley State Park, and the National Oceanic and Atmospheric Administration (NOAA) started a multi-year effort to restore ecological connectivity in the Patapsco River watershed. Although two dams (Union and Simkins) were removed in 2010, Bloede Dam, built in 1907, remained as the most downstream barrier for fish migrating up the Patapsco River (Fig 1B). Bloede Dam was located approximately 18 km (11 mi) upstream of the Chesapeake Bay and was designed as a flat slab buttress hydroelectric dam with a 10 m (34 ft) high spillway spanning 67 m (220 ft) across the river valley [34]. A concrete Denil-type fish ladder was added to the right abutment of the dam in 1992. In September 2018, Maryland DNR breached Bloede Dam and began the process of dismantling the dam structure and full-scale restoration of the riverbanks. All dam removal and restoration work was completed in May 2019. During the construction period, a temporary causeway was installed just upstream of the dam location, which may have served as a potential fish passage barrier. After full restoration was complete (Fig 1C), this dam removal effectively restored access to approximately 103 km (64 mi) of free-flowing freshwater habitat for anadromous fish species along the main stem and tributaries [34]. Monitoring of fish habitat use and migration occurred from 8.3 km (5 mi) below the former Bloede Dam as far as 12.9 km (8 mi) upstream of Daniels Dam, but did not extend as far as Liberty Dam, the last major artificial obstruction that intersects the North Branch of the Patapsco River. This study was conducted under proposal numbers SERC-03-01-2013, SERC-12-06-2016, and SERC-2020-0131-01 which received written approval by the Smithsonian Environmental Research Center Animal Care and Use Committee.

### Environmental DNA sampling

Environmental DNA is an emerging, non-invasive monitoring tool used to detect river herring presence via naturally shed DNA. Sampling sites were categorized into three groups according to their geographic location relative to the Bloede Dam site including “Downstream” of Bloede Dam (n = 8), “Restored” sites between Bloede Dam and Daniels Dam (n = 5), and “Above Daniels” sites upstream of Daniels Dam (n = 3; Fig 1). Water samples (~800 ml) were collected in 1 L Nalgene bottles. Before sample collection, bottles and caps were rinsed with a 10% bleach solution. The bottles were also autoclaved no more than 5 days prior to sampling and stored in a sealed cabinet with other clean bottles or in bleached collection coolers. Once bottles were autoclaved, they were not opened until sample collection. If bottles were new, they were only autoclaved prior to sample collecting. After collection, samples were frozen in a non-defrosting freezer until further analysis. A total of 490 eDNA samples, including control samples, were collected across the 16 sample sites between 2015 and 2021 (Table 1). Of these samples, 189 were collected from Downstream sites, 117 from Restored sites, and 97 from Above Daniels sites. Duplicate samples were also collected at all sites in 2019 (n = 109 out of 140 non-control samples), 2020 (n = 52 out of 105), and 2021 (n = 54 out of 102) (Table 1). One control sample was collected for each sampling event (n = 39) to show that samples were not contaminated during the collection, transport, or storage processes.

**Table 1:** eDNA sampling effort across Patapsco River study sites from 2015 to 2021.

	Site Name	Year						
		2015	2016	2017	2018	2019	2020	2021
**Above Daniels**	ad3. Marriottsville North Branch	1	1	1	1	2	2	2
	ad2. Marriottsville South Branch	1	1	1	1	2	2	2
	ad1. Daniels Dam Up	ns	ns	ns	ns	2	2	2
**Restored**	r5. Daniels Dam Down	ns	ns	ns	ns	2	2	2
	r4. Alberton	1	1	1	1	2	ns	ns
	r3. Old Frederick Road	1	1	1	1	2	2	2
	r2. Ellicott City	ns	ns	ns	ns	2	2	2
	r1. Illchester Rd	ns	ns	ns	ns	2	2	2
**Downstream**	d8. Orange Grove	ns	ns	ns	ns	2	2	2
	d7. Mainstem at Rockburn	1	1	1	1	2	2	2
	d6. Rockburn Run	1	1	1	1	2	2	2
	d5. DIDSON-PT	ns	1	ns	1	2	2	2
	d4. Deep Run Hanover Rd	1	1	1	1	2	ns	ns
	d3. Deep Run Furnace Rd	1	1	1	1	2	2	2
	d2. Stoney Run	1	1	1	1	2	ns	ns
	d1. Food Bank	ns	ns	ns	ns	2	2	2

Sites are arranged in order from farthest upstream to lowest downstream. “1” = single sample, “2” = duplicate sample, “ns” = not sampled. Reference locations for each site by code (d1-8, r1-5, ad1-3) on Fig 1; total sampling effort by site in S2 Fig.

A river herring-specific quantitative PCR (qPCR) molecular beacon assay was used to identify river herring DNA sequences following established procedures [35]. Briefly, water samples were thawed and filtered using 47 mm diameter Whatman cellulose nitrate filters with 1.0 μm pore size. DNA was extracted with the Omega Biotek EZNA Water kit following manufacturer’s instructions or with a CTAB Chloroform-Isoamyl extraction procedure [36]. qPCR was conducted on sample extracts in triplicate, and samples with at least two out of three triplicates with cycle quantification (Cq) values below 39 were considered positive eDNA detections (i.e., river herring presence) (n = 402 out of 451 non-control samples). This Cq value has previously been established as the conservative threshold for true eDNA detections for river herring assays [35]. For samples with positive river herring detection, species-level identification of alewife and blueback herring was determined via Sanger sequencing [35]. The relative ratio of alewife to blueback herring DNA in each sample was estimated based on the relative peak height ratios at a species-diagnostic SNP produced by QSVAnalyser [37] and was used to calculate the number of eDNA copies per liter for each species. Copy numbers from qPCR amplification were then adjusted for each sample based on the filtered water volume, calculated as the number of mtDNA copies per liter of water sampled. Duplicate samples were collected from the sites from 2019 to 2021, so copy numbers were averaged across all replicates collected at the same site and time prior to analysis.

### Ichthyoplankton sampling

Ichthyoplankton sampling was conducted to assess spawning activity in the Patapsco River. Surveys of river herring eggs were conducted simultaneously with the collection of eDNA water samples at the “Downstream” (n = 8), “Restored” (n = 5), and “Above Daniels” (n = 3) sites (Fig 1, S1 Fig), following established protocols and standard methods previously used by Maryland DNR [38]. A stationary 46 cm x 30 cm plankton drift net with 500 μm mesh and a 200 mL cod end was deployed for 5 minutes per sample. In total, 284 samples were collected over the course of this study. Water velocity measurements from a flowmeter (JDC Electronics Flowatch) were used to estimate sample volume at each location. Eggs and larvae were retrieved, counted, and identified under a dissecting microscope. It is not possible to visually distinguish between alewife and blueback herring eggs, or between river herring and hickory shad (*Alosa mediocris*), due to morphological similarities at the early developmental stages.

Prior to statistical analysis, a qualitative lower threshold was established for the egg count data to account for potential sampling error (methods similar to [39]). Observations with two or fewer eggs at any site were set as zero for “non-detection” (n = 34) and thus excluded from the average. The lower detection threshold would account for potential sampling cross-contamination, where residual eggs may not be thoroughly cleaned or removed from the net between new sampling events at different sites. Biological significance also informed the threshold, as even a single spawning female river herring can release hundreds of thousands of eggs into the water column [40].

Egg abundance was converted to catch per unit effort (CPUE) across the dataset, standardized as number of eggs 100 kL^-1^ of water. Calculating the volume of water passing through the collection net accounted for measured flow (cm s^-1^) at each site/sample, net area (cm^2^), and collection time (s). Normalized CPUE was rounded to the nearest egg to obtain an integer count value for subsequent models. Mean egg abundance was then compared among the sampling sites.

### Electrofishing sampling

Adult river herring were collected during boat electrofishing surveys at six sites on the mainstem of the Patapsco River from 2015 through 2021. Three sites were located downstream of Bloede Dam (“Downstream-electrofishing”) and three sites were located upstream (“Restored-electrofishing”; S2 Fig). Two of the Downstream-electrofishing sites (#591 and #592) were sampled for the full duration of this study (2015–2021). The remaining Downstream-electrofishing site (#593), located just below Bloede Dam in the dam’s tailrace, was not sampled after 2018 because the site converted from a pool to rapids following the dam removal and was no longer safely boatable. Boat electrofishing was not conducted above Bloede Dam (#595, #596, and # 597) between 2015 and 2018, because prior monitoring conducted from 2011 to 2014 in the dam pool and at the fish ladder exit indicated no river herring passage [31]. Surveys were not collected at these Restored-electrofishing sites in 2019 due to mechanical issues with the electrofishing boat. Field activities in 2020 were severely impacted by COVID-19 restrictions, limiting the number of sampling events at both Downstream- and Restored-electrofishing sites. All electrofishing sites (barring # 593) were fully sampled in 2021.

Electrofishing surveys were conducted weekly at each site during the river herring spawning season (March through May), with a target of ten sampling visits per site per year. Due to COVID-19 restrictions, we were only able to sample the Downstream-electrofishing sites twice (two events in March) and the Restored-electrofishing sites five times (two events in March and three events in May) in 2020. Electrofishing at all sites was performed from a small boat while moving downstream, with total electrofishing time, fish species present, and abundance of river herring recorded for each site. Using the recorded electrofishing time and the numbers of river herring caught, relative abundance (fish collected per hour of electrofishing) was estimated for each species. Collected river herring were identified to species and total length and sex (determined based on the presence of milt or eggs, when possible) were recorded for each individual.

### Passive integrated transponder (PIT) telemetry

Habitat use and spawning migration movement by individual adult fish was assessed using passive integrated transponder (PIT) telemetry. PIT tags are passive radio tags that allow the tracking of individual adult fish movement during their migration [27, 41]. River herring were captured via boat electrofishing from within the study system and each fish was identified to species, measured (total and fork length) and sexed (determined based on the presence of milt or eggs, when possible). All fish were surgically implanted with a 23 mm × 3.65 mm HDX+ PIT tag (Oregon RFID, Portland, Oregon, USA) following established intraperitoneal tagging methods [42]. In short, a small incision was made just posterior to the pelvic fin, roughly three scale rows forward of the ventral midline. A PIT tag was then immediately inserted manually and the fish placed into an aerated live well. Once tagged fish returned to normal swimming behavior they were released back in the river. The scalpel width (#15 blade, 3.75 mm) was slightly larger than the tag width (3.65 mm) and closed neatly after tag implantation; thus, negating the use of sutures. Surgical handing times were short, generally less than 30 s per fish.

Tagged fish were tracked from 2016 to 2021 using multiple PIT tag antenna/reader systems (Oregon RFID, Portland, Oregon, USA) deployed in the Patapsco River. The configuration of all antennas (comprising 4-gauge fine strand copper wire) was a pass-over loop that stretched along bottom of the entire stream width (~ 10 m). The antenna shape and integrity were maintained by 3/16 in chain attached to the antenna wire and 69.5 cm sections of 1.25 in schedule 40 PVC pipe filled with cement spaced at roughly 1.5 m intervals attached to both sides of the chain in the center of the loop. We deployed antennas at three sites: “Downstream-antenna #1”, “Downstream-antenna #2”, and “Restored-antenna” (S3 Fig). For three years pre-removal (2016–2018), only the two antennas located downstream of Bloede Dam (Downstream-antenna #1 and #2) were deployed. In 2019 and 2021, all three antennas were deployed with the Restored-antenna located upstream of the former Bloede Dam location. River herring were not tagged or tracked in 2020 due to COVID-19 restrictions on field activities. PIT tag detections were automatically logged with a timestamp by tag readers. For analysis, detections for each unique tag at any given antenna were aggregated by date.

### Statistical analysis

Statistical analyses were conducted in R version 4.1.1 using generalized linear mixed models (GLMM) in the *glmmTMB* R package [43, 44]. We used a hurdle model framework [45] to examine differences in river herring eDNA and egg presence-absence and concentration between pre- and post-dam removal periods across the three groupings of habitat sites (Downstream, Restored, Above Daniels). Differences in the presence-absence of eDNA and eggs pre- and post-dam removal were assessed using logistic regressions with logit link functions, dam removal as a fixed effect and sampling site as a random effect. Differences in eDNA concentration and egg CPUE pre- and post-dam removal were assessed using linear models with gaussian error structures, identity link functions, dam removal as a fixed effect and sampling site as a random effect. In these analyses, both eDNA concentration and egg abundance were log-transformed and only included data with concentrations greater than zero. The three habitat site groupings were examined independently for both the presence-absence and concentrations of eDNA and eggs. Post-hoc pairwise comparisons were conducted using the *emmeans* R package [46, 47]. To generate model estimates for presence-absence, probabilities and standard errors were back-transformed from the logistic regression to the response scale. Model assumptions and fit for all models were assessed using the *DHARMa* package [48]. All analyses of eDNA were species-specific for alewife and blueback herring, while eggs were collectively analyzed since the species could not be distinguished.

In addition to the primary analyses comparing pre- and post-dam removal, we compared the presence-absence and concentration of eDNA between the two species, alewife vs. blueback herring, using a similar hurdle model framework. Differences in the presence-absence of eDNA were assessed using logistic regressions with a logit link function, species as a fixed effect, habitat group as a random effect, and sampling site as a random effect nested within habitat group. Differences in the log-transformed concentration of eDNA were assessed using linear models with gaussian error structures, identity link functions, species as a fixed effect, habitat group as a random effect, and sampling site as a random effect nested within habitat group. This model included only data with concentrations greater than zero. Only eDNA was compared between species as eggs could not be distinguished.

The seasonal movements of PIT tagged fish were assessed by estimating the percentage of tagged fish that were detected at the Downstream-antenna #1 that were then also detected at either the Downstream-antenna #2 or Restored-antenna (following [26]. Quantifying detections at multiple antennas in this way reduces the extent to which external factors (i.e., tagging stress, natural mortality, other sources of tag loss) influence the results. Collections of river herring during boat electrofishing surveys were used to confirm the presence of adult alewife and blueback herring at sites downstream and upstream of the Bloede Dam site.

## Results

### Distribution and concentration of alewife and blueback herring eDNA

A total of 451 non-control eDNA samples were collected and processed across 16 sites between 2015 and 2021, with 120 total samples positive for river herring eDNA. After accounting for duplicates, 28% of all samples produced positive river herring eDNA detections. All control samples (n = 39) were negative for river herring eDNA. Duplicate samples most consistently produced positive detections in both samples at Downstream sites (68% of positive detections), whereas only 17% of positive detections for Restored sites were positive in both duplicate samples. There were 13 samples where only alewife DNA was detected and 42 samples where only blueback herring DNA was detected.

**Fig 2:**
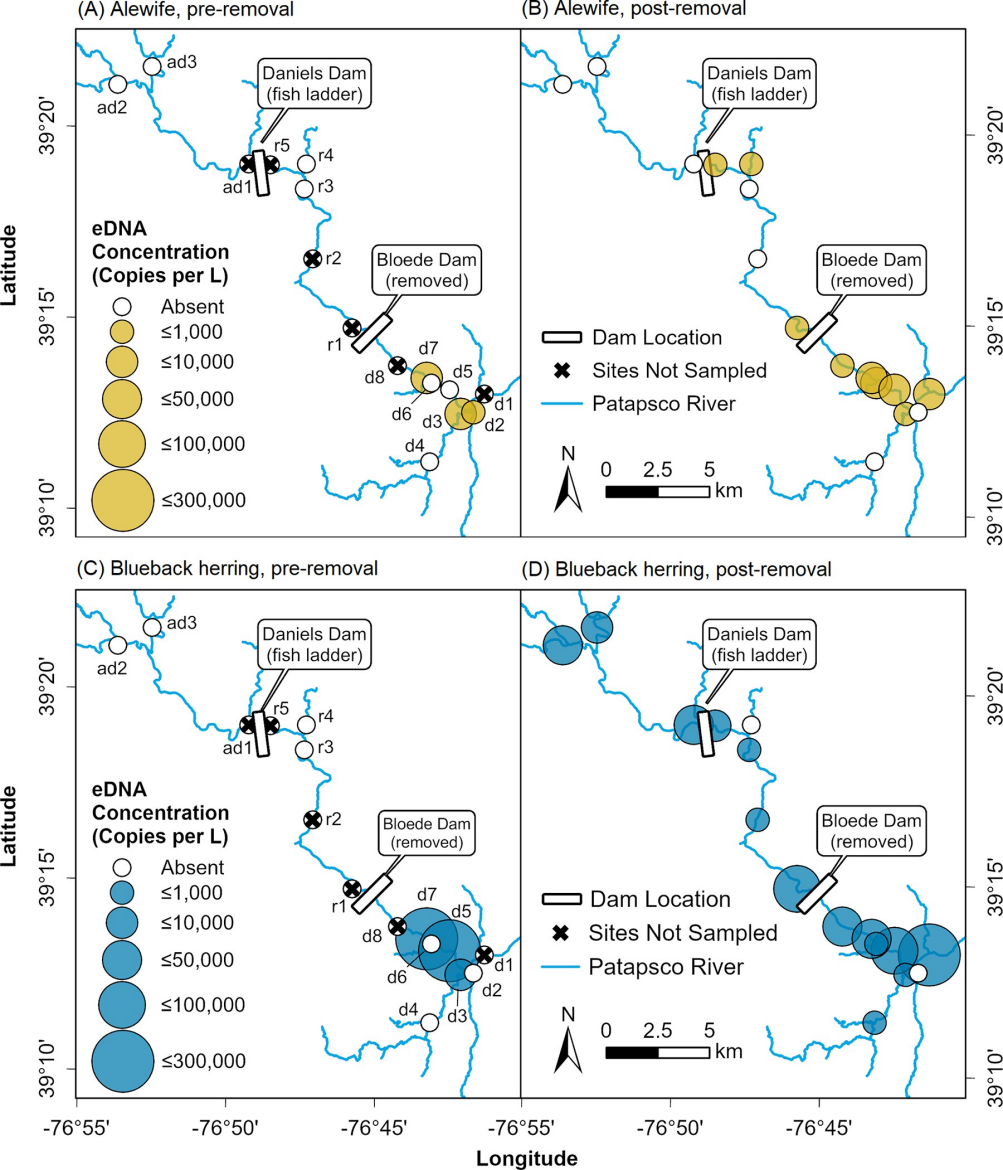
Map of positive river herring eDNA detections in the Patapsco River pre- and post- removal of Bloede Dam. Concentrations of alewife eDNA before (A) and after (B) and blueback herring eDNA before (C) and after (D) the removal of Bloede Dam. Size of the points are relative to the mean number of mtDNA copies/L.

**Fig 3:**
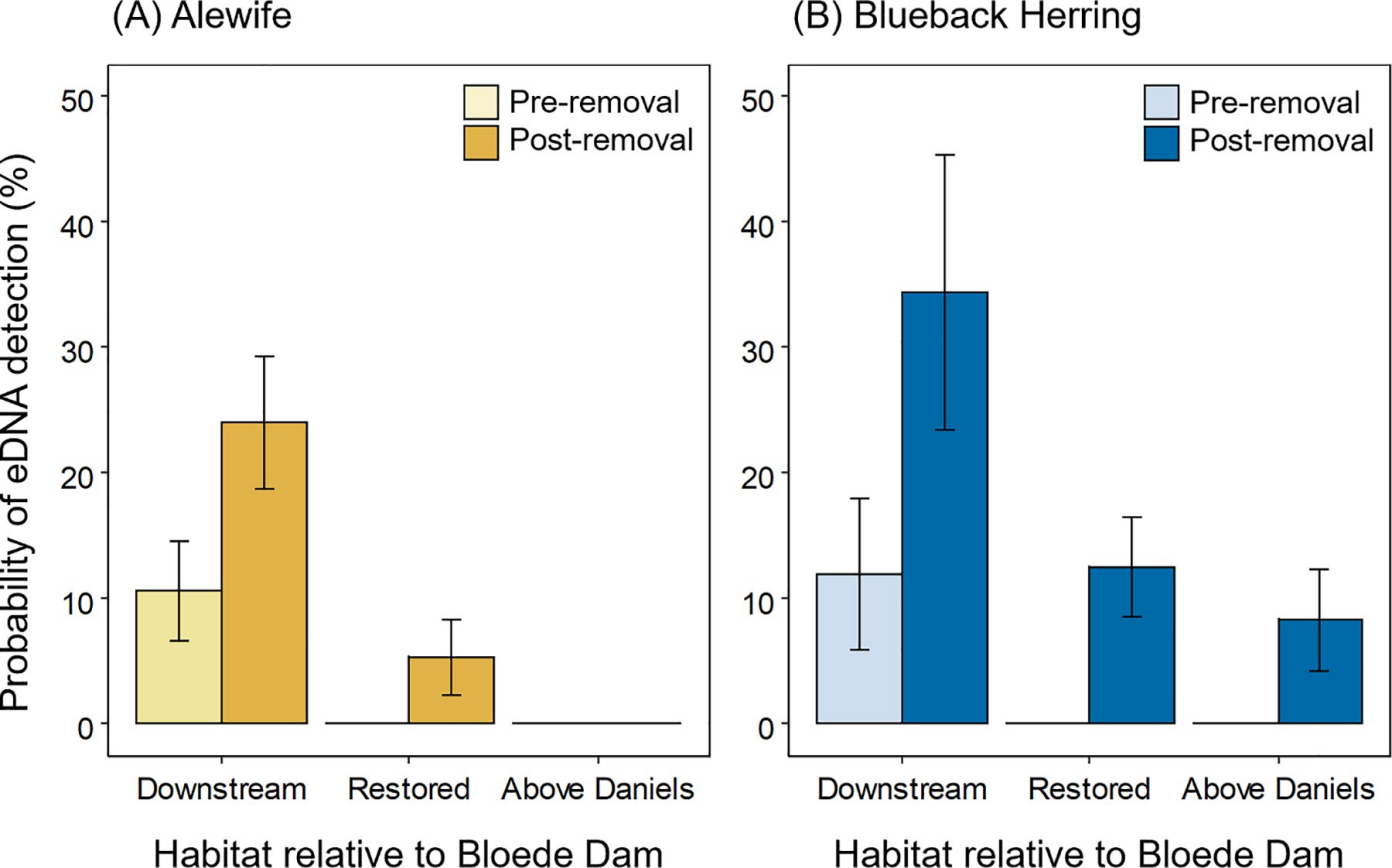
Probability of detected river herring eDNA in the Patapsco River pre- and post- removal of Bloede Dam. Estimated detection probability of alewife (A) and blueback herring (B) eDNA at Downstream, Restored, and Above Daniels sites in the Patapsco River pre- and post- removal of Bloede Dam. Least squares means and standard errors are back-transformed from the logistic regression to the response scale.

Both alewife and blueback herring eDNA was detected at sites upstream of Bloede Dam after the dam’s removal, but not before (alewife: Fig 2A and 2B, blueback herring: Fig 2C and 2D). Post-removal, the probability of detecting alewife eDNA at Restored sites increased from 0% to 5.4 ± 3.0%, while the probability of detecting blueback herring eDNA at Restored sites increased from 0% to 12.5 ± 4.4% (estimate ± SE; Fig 3). In 2019, during the spring migration immediately after the removal of Bloede Dam, eDNA from both species was detected at the site farthest upstream in the restored segment, immediately below Daniels Dam. In addition to increased eDNA detections at Restored sites upstream of Bloede Dam, detections of eDNA increased at Downstream sites for both species. The chance of detecting alewife eDNA increased from 10.6 ± 3.9% to 24.0 ± 5.3% (odds ratio = 2.67 ± 1.27, *t*_146_ = 2.06, *p* = 0.041), while the chance of detecting blueback herring eDNA increased from 12.0 ± 6.0% to 34.4 ± 10.9% (odds ratio = 3.87 ± 1.84, *t*_146_ = 2.84, *p* = 0.005).

**Fig 4:**
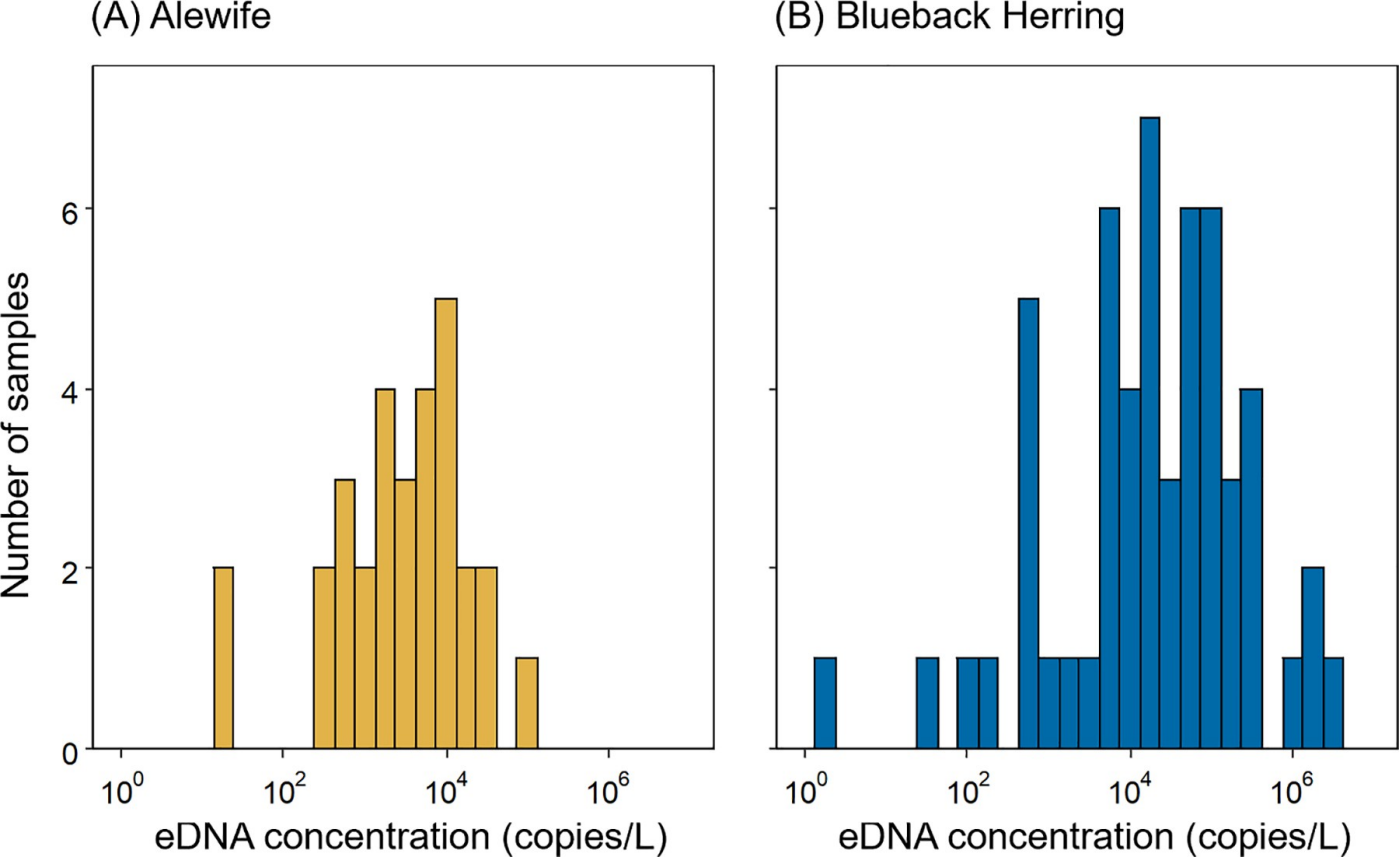
Distribution of river herring herring eDNA concentrations. Concentrations of eDNA for positive detections of alewife (A) and blueback herring (B) from 2015 to 2021 sampling in the Patapsco River. Concentrations are normalized as number of mtDNA copies/L.

**Fig 5:**
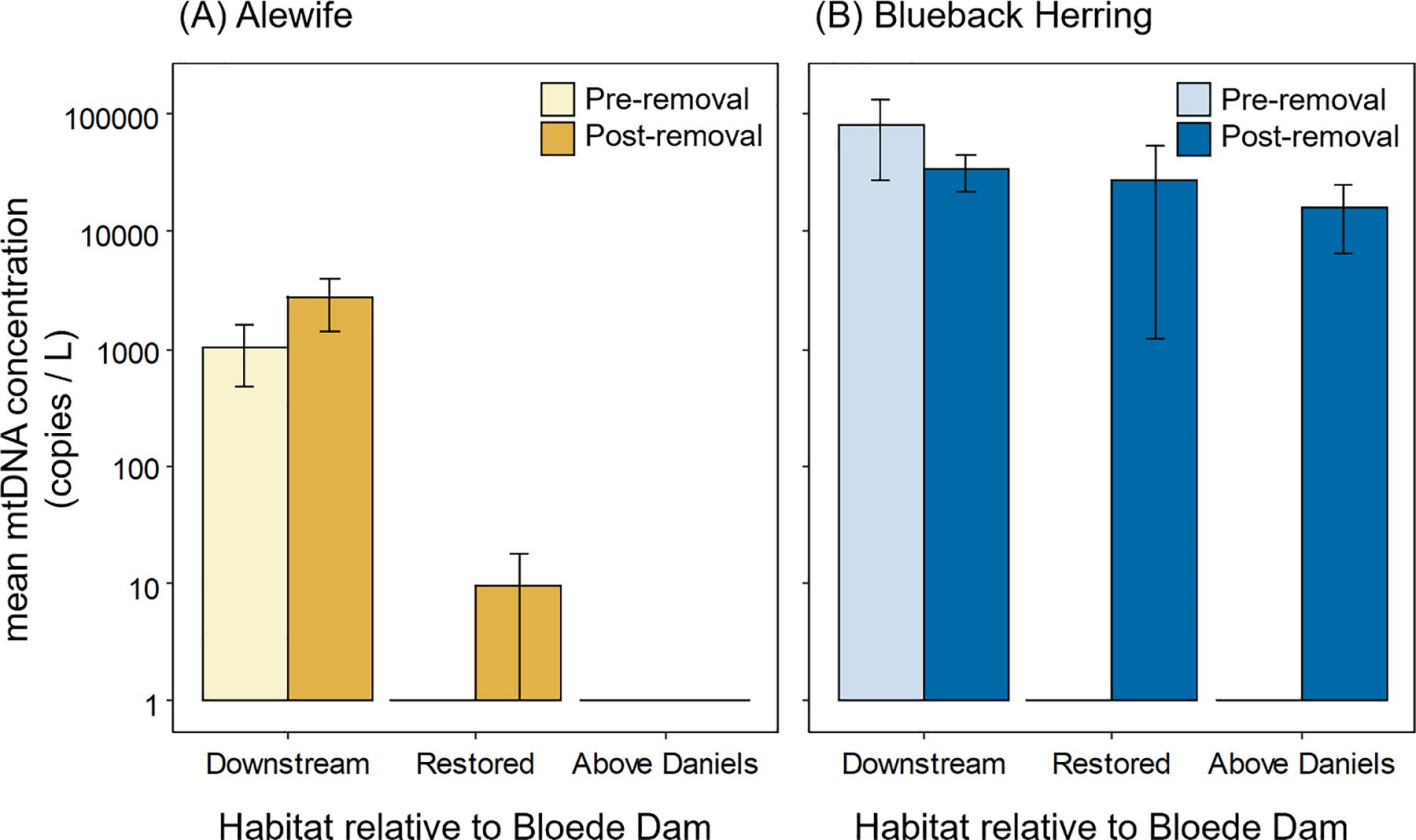
Concentration of river herring eDNA in the Patapsco River pre- and post- removal of Bloede Dam. Mean concentration of alewife (A) and blueback herring (B) eDNA at Downstream, Restored, and Above Daniels sites in the Patapsco River pre- and post- removal of Bloede Dam. Concentrations are normalized as number of mtDNA copies/L. Means include zeros and are shown on a log scale. Bars show standard errors.

We compared changes in river herring eDNA concentrations pre- and post-dam removal for Downstream sites only, because there were no positive detections at Restored and Above Daniels Dam sites pre-removal. In samples with positive detections, concentrations of alewife eDNA ranged from 20 to 103,384 copies L^-1^ (9,812 ± 3,555 copies L^-1^, mean ± SE; Fig 4A). For blueback herring, concentration from positive detections ranged from 2 to 2,685,977 copies L^-1^ (184,846 ± 68,049 copies L^-1^; Fig 4B). While the probability of detecting eDNA increased across all sites for both alewife and blueback herring post-dam removal, there were not statistically significant changes in relative eDNA concentrations at Downstream sites. Pre-removal, a mean of 1,041 ± 559 copies L^-1^ (mean ± SE, Downstream sites only, zeros removed) of alewife eDNA were collected at Downstream sites while 2,712 ± 1,320 copies L^-1^ were collected post-dam removal (estimate = 0.29 ± 0.64, *t*_23_ = 0.456, *p* = 0.653; Fig 5A). For blueback herring, a mean of 80,323 ± 52,927 copies L^-1^ were collected pre-removal and 33,633 ± 11,526 copies L^-1^ were collected post-removal (estimate = 0.91 ± 0.87, *t*_41_ = 1.05, *p* = 0.299 Fig 5B).

Finally, we compared the changes in eDNA detectability and concentration between alewife and blueback herring. Prior to the removal of Bloede Dam, there were no statistically significant differences between alewife and blueback herring in the presence-absence (odds ratio = 0.63 ± 0.35, *t*_212_ = -0.82, *p =* 0.414) or concentration (estimate = -1.83 ± 1.28, *t*_12_ = -1.44, *p =* 0.177) of eDNA. Post-removal the estimated probability of detecting blueback herring eDNA was 15.79 ± 8.08% compared to 6.47 ± 3.85% for alewife (odds ratio = 0.37 ± 0.12, *t*_346_ = -3.18, *p =* 0.002). The concentration of eDNA was also greater for blueback herring, 108,116 ± 51,296 copies L^-1^ (mean SE, all sites, excluding zeros) compared to 9,811 ± 4,502 copies L^-1^ for alewife (estimate = -1.88 ± 0.59, *t*_63_ = -3.18, *p =* 0.002). In addition, blueback herring eDNA was detected in three samples at sites Above Daniels Dam (Marriottsville North and Marriottsville South Branch) in 2021, increasing the chance of detection from 0% to 8.3 ± 4.6% (Fig 2D). Alewife eDNA was not detected at any sites Above Daniels Dam.

### Distribution and concentration of eggs

**Fig 6:**
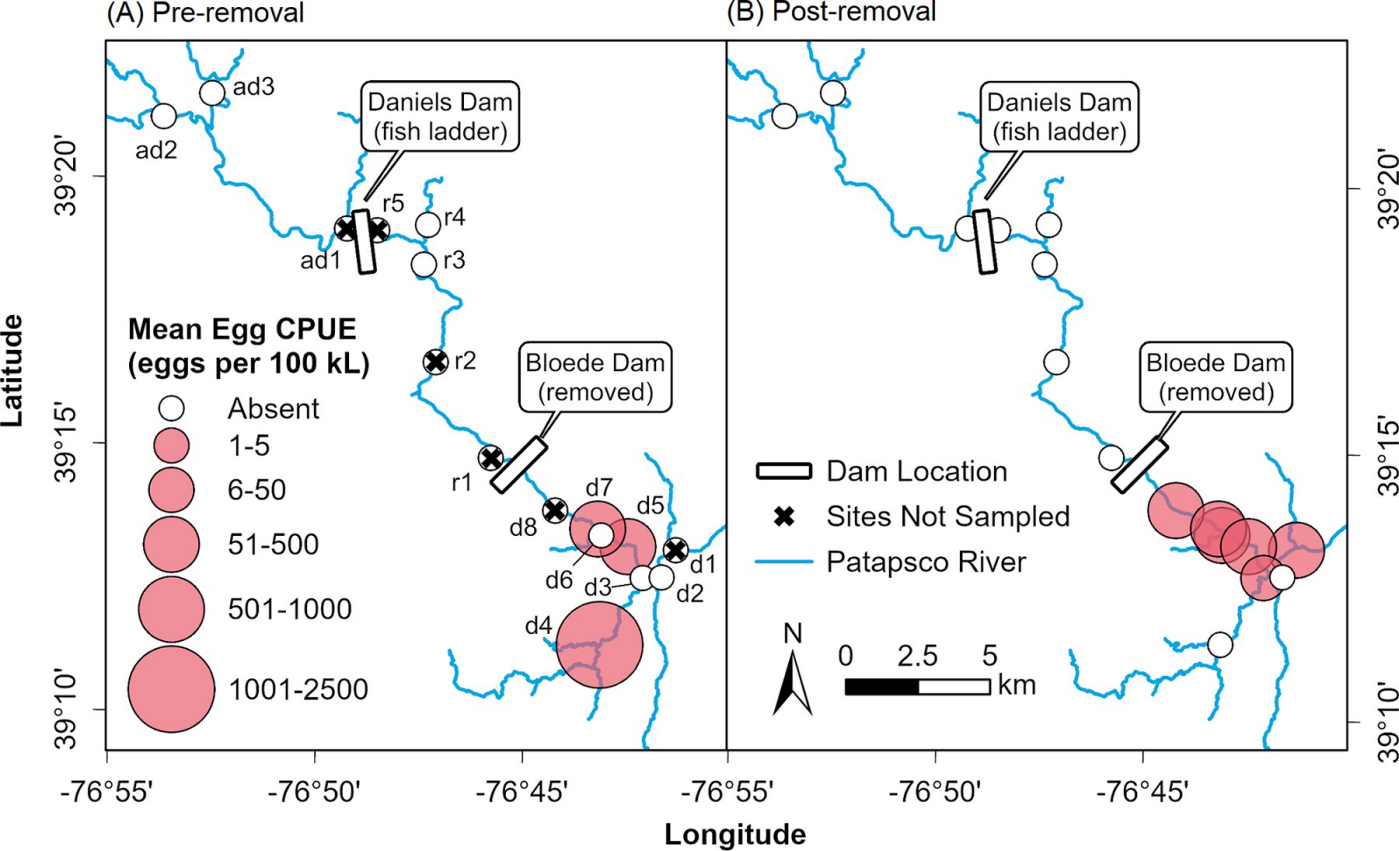
Map of distribution and abundance of river herring spawning activity in the Patapsco River pre- and post-removal of Bloede Dam. Abundance of river herring eggs collected pre-removal (A) and post-removal (B) of Bloede Dam. Catch per unit effort is standardized as number of eggs/100 kL.

**Fig 7:**
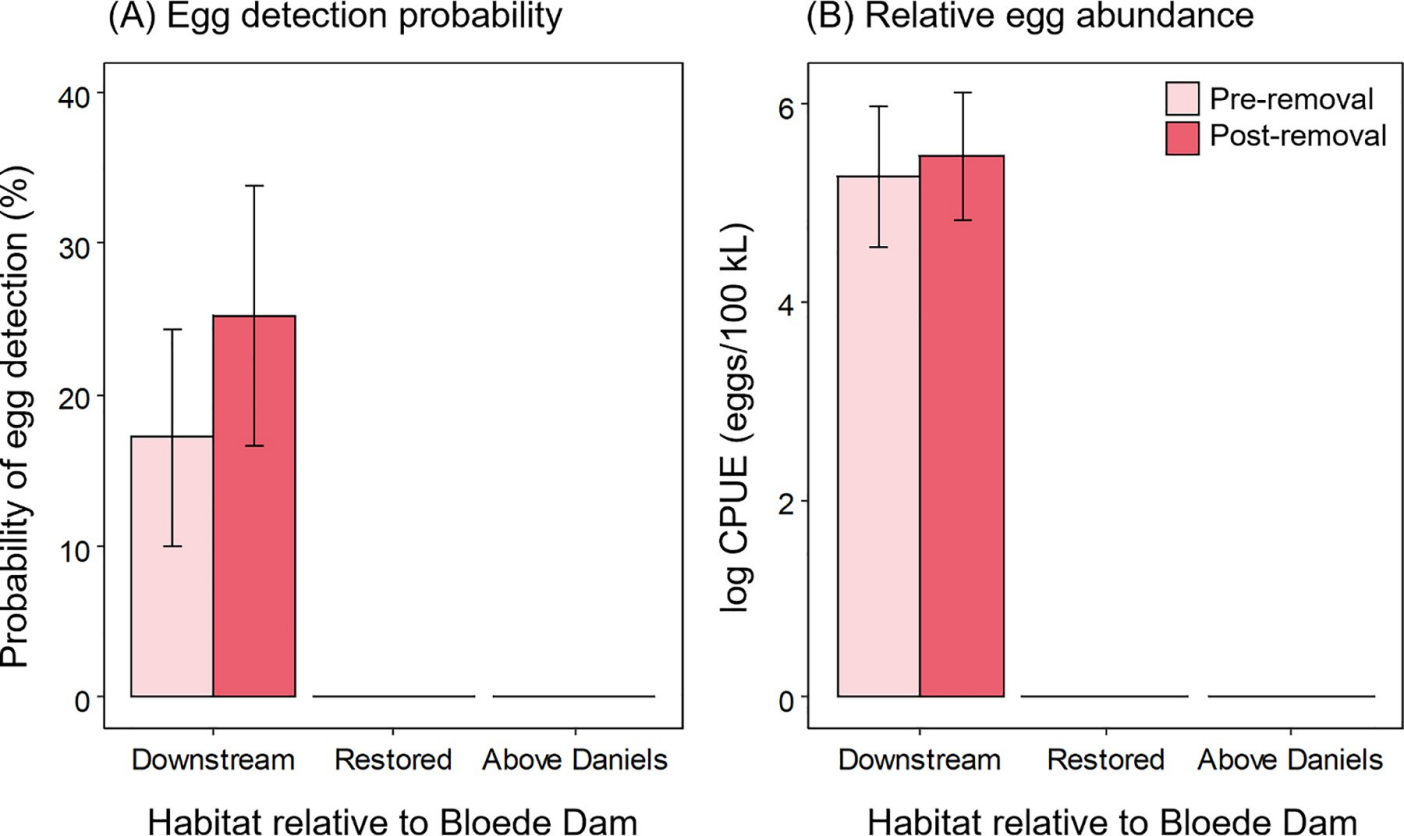
Probability and abundance of river herring eggs pre- and post-removal of Bloede Dam. Estimated detection probability (A) and relative abundance (B) of river herring eggs at Downstream, Restored, and Above Daniels sites in the Patapsco River pre- and post- removal of Bloede Dam. Egg detection probabilities are back-transformed from the logistic regression to the response scale. Values are the least squares means and bars show standard error.

River herring eggs were not detected upstream of the Bloede Dam site either before or after the dam’s removal (Fig 6). Overall, there was a 14% positive detection rate (41 out of 284 samples) for eggs across all sites and years. Pre-removal, river herring eggs were detected at three out of five (60%) sampled sites in the river downstream of the dam site. Post-removal, eggs were detected at six out of seven (86%) sampled sites in the downstream segment. Notably, in 2019, eggs were detected at two Downstream sites (Orange Grove and Rockburn Run) sites where eggs had not been detected pre-removal. The probability of detecting river herring eggs at downstream sites increased from 17.2 ± 7.2% (mean ± SE) pre-removal to 25.2 ± 8.6% post-removal (odds ratio = 1.62, *t*_138_ = 1.06, *p* = 0.289; Fig 7A). The mean concentration of eggs was higher pre-removal (2,055 ± 1,922 eggs 100 kL^-1^, mean ± SE, Downstream sites only, zeros removed) compared to post-removal (390 ± 110 eggs 100 kL^-1^). The higher pre-removal mean value was skewed by a single sample where a large number of eggs were collected at low discharge (Deep Run in 2015). On a log-scale, the concentrations of eggs pre- and post-dam removal were not statistically different (estimate = 0.20, *t*_36_ = -0.35, *p* = 0.728; Fig 7B).

### Electrofishing surveys

Alewife and blueback herring were regularly collected in electrofishing samples at the three Downstream-electrofishing sites pre- and post-dam removal, except in 2020 when the survey was not fully conducted (Table 2). Adult alewife and blueback herring were both collected at Restored-electrofishing sites upstream of the former Bloede Dam for the first time in 2021. A single male alewife was collected at the first site upstream of the former dam (#595) on March 26, 2021, and a single male blueback herring was collected at the second site upstream of the former dam (#596) on May 13, 2021 (S4 Fig).

**Table 2:** Number of adult alewife (AW) and blueback herring (BB) detected in boat electrofishing surveys in the Patapsco River.

	Downstream-electrofishing sites						Restored-electrofishing sites					
Year	#591		#592		#593		#595		#596		#597	
	AW	BB	AW	BB	AW	BB	AW	BB	AW	BB	AW	BB
2015	128	42	20	4	21	74	-	-	-	-	-	-
2016	115	83	24	137	88	202	-	-	-	-	-	-
2017	36	68	12	29	8	26	-	-	-	-	-	-
2018	45	261	9	164	21	31	-	-	-	-	-	-
2019	75	66	9	42	-	-	-	-	-	-	-	-
2020	0	0	1	0	-	-	0	0	0	0	0	0
2021	91	4	86	58	-	-	1	0	0	1	0	0

Electrofishing surveys were conducted from 2015–2021 from three sites downstream of Bloede Dam (591,592,593) and three sites upstream of Bloede Dam in the restored section of the river (595, 596, 597). The survey was only partially conducted in 2020. Site 593 was not surveyed after 2018 and Restored sites were not surveyed before 2020. The dotted horizontal line represented the removal of Bloede Dam.

### PIT telemetry

There is no evidence of tagged fish moving upstream into restored habitats after Bloede Dam was removed, since we did not detect tagged fish of either species at the Restored-antenna in 2019 and 2021. In total, 640 alewife (261 female and 379 male) and 1122 blueback herring (409 female, 709 male, and 4 unspecified) were tagged during this study. Approximately 25% of either tagged alewife (n = 159) and tagged blueback herring (n = 271) were detected at any antenna at least once over the entire sampling period. Prior to the removal of Bloede Dam, tagged adult river herring were consistently detected at the two PIT tag antennas downstream of the dam site (Table 3). Less than 10% of tagged alewife or blueback herring detected at the Downstream-antenna #1 were also detected at Downstream-antenna #2. Post-removal in 2019, 19% of alewife and 14% of blueback herring detected at the Downstream-antenna #1 also reached Downstream-antenna #2, representing the greatest proportion of individual fish detected migrating into this midstream reach of the river below the Bloede Dam site. However, in 2021, only between 3–7% of tagged fish detected at the Downstream-antenna #1 reached Downstream-antenna #2. During the pre-removal period (2016 to 2018), the number of tagged blueback herring individuals detected at the Downstream-antenna #1 was consistently greater than that of alewife.

**Table 3:** Number of tagged alewife (AW) and blueback herring (BB) detected at PIT receiver antenna sites (Downstream-antenna #1, Downstream-antenna #2, and Restored-antenna).

	Downstream-antenna #1		Downstream-antenna #2				Restored-antenna			
Year	BB count	AW count	BB count	%	AW count	%	BB count	%	AW count	%
2016	7	0	4	0	0	0	-	-	-	-
2017	56	29	7	10.7	1	<0.1	-	-	-	-
2018	157	59	13	2.6	4	5.1	-	-	-	-
2019	43	43	8	14.0	15	18.6	0	0	0	0
2021	15	30	1	6.7	2	3.3	0	0	0	0

PIT tag receivers were installed and collected data from 2016 to 2021, excluding 2020. Percentage represents the proportion of fish detected at the downstream site that were also detected at the midstream or upstream site. The Ellicott City antenna upstream of Bloede Dam was only installed in 2019.

## Discussion

### Electrofishing and eDNA samples suggest recovery of spawning habitat use in response to dam removal

We integrated four monitoring methods (eDNA, eggs, electrofishing, PIT tags) to record rapid expansion and recovery of river herring spawning habitat in the Patapsco River following a large-scale dam removal. Overall, we confirmed our prediction that river herring would access restored habitats within three years of the dam removal. Prior to the removal of Bloede Dam, neither eDNA, eggs, nor adult river herring were detected upstream of the dam despite the presence of a fish ladder. This result was consistent with earlier electrofishing monitoring efforts by Maryland DNR [31]. In the first year post-dam removal, river herring eDNA was detected upstream of the former dam site, indicating expanded fish presence in restored habitat even before full restoration work at the site was completed. Electrofishing surveys further confirmed river herring presence in the restored reach in the third year post-removal, 2021, with the capture of one adult alewife and one adult blueback herring. River herring can be particularly good at reoccupying new spawning habitats if stream reaches are well connected; straying rates of over 30% have been observed among sites within a river system [49]. Our results are thus consistent with previous studies in other rivers that reported rapid expansion of spawning habitat use by alosines when dams are removed [12, 15, 34, 50].

Our results further indicated the spatial extent of restored habitat access for river herring following dam removal. In 2021, blueback herring eDNA was present upstream of Daniels Dam on two separate sampling days just before the fish ladder closed in May 2021 to prevent the spread of invasive northern snakehead in the Patapsco River. Prior to 2018, passage efficiency at the Daniels Dam Denil-type fish ladder was impossible to measure because river herring were not passing Bloede Dam [31]. The positive detection and high concentrations of blueback herring eDNA upstream of Daniels Dam indicates that the fish are not only using newly restored habitat immediately beyond Bloede Dam, but are also capable of migrating past Daniels Dam to some degree as long as the fish ladder remains open. Thus, by removing Bloede Dam, even more habitat than originally estimated may now be available to spawning river herring.

Although the presence of river herring eDNA and adult fish in electrofishing surveys upstream of the former Bloede Dam indicated successful use of restored habitat, no PIT tagged alewife or blueback herring were detected by the Restored-antenna deployed upstream of the former dam. Detections of PIT tags reflected a trend of decreasing abundance moving upstream across the entire monitoring period, where an average of 12% of tagged river herring at the Downstream-antenna #1 also reached Downstream-antenna #2. This result is consistent with the decreasing detection probability of both alewife and blueback herring eDNA moving from Downstream to Restored and Above Daniels sites. Previous studies have demonstrated similar “truncated” upstream distribution patterns of anadromous fishes in response to stream restoration [10, 50]. For example, a linear decline in PIT tagged American Shad (*Alosa sapidssima*) abundance in the Little River, North Carolina, was observed after a dam removal, with the highest proportion of detected fish remaining in downstream reaches [26]. Overall, longer term and more extensive telemetry studies in the Patapsco River are needed to track the movement of individual tagged fish into restored reaches of the river.

### No evidence of spawning in restored habitats from egg samples

Despite increased presence of river herring eDNA in the Patapsco River after Bloede Dam was removed and the detection of adult fish above the dam site, egg count data did not provide evidence of spawning activity in the newly accessible habitat. There are few prior studies that sample ichthyoplankton to monitor anadromous fish spawning after dam removals, especially for alosines on the East Coast of the United States. Previous studies examining the presence of eggs and larvae after dam removals tend to focus on changes over longer timescales, after several years post-restoration [25, 50]. Thus, similar longer-term sampling in the Patapsco River is needed to identify potential positive impacts of the dam removal on spawning activity and eventually population size.

The lack of eggs detected upstream of the Bloede Dam site, however, does not necessarily indicate a true absence of reproductive activity in the restored habitats post-removal. Each of our other data types suggests that far fewer fish are using the Restored sites than Downstream sites, so it is possible that spawning is taking place and the concentration of eggs at Restored sites is so low that we didn’t detect them. In addition, our positive eDNA results may indicate spawning in the restored section of the river, as eDNA can be derived from biological material shed during any life stage of the fish, including eggs, larvae, or other maternal tissues expelled during broadcast spawning [35, 51]. In fact, observed increases in fish eDNA in ambient water may overlap with reproduction timing and spawning events [52]. More frequent ichthyoplankton sampling at longer sample durations may aid in documenting whether spawning is occurring in the restored reaches of the river.

### eDNA samples reveal species-specific responses to the dam removal

Our eDNA results suggest alewife and blueback herring may have had different responses to the dam removal. While the presence of eDNA increased for both species, use of restored habitat was greater for blueback herring. Pre-removal, we observed no differences in the presence and concentration of eDNA between the species. Post-dam removal, there was approximately an 84% greater probability of detecting blueback herring eDNA across all sampling sites compared to alewife and the mean concentrations of blueback herring eDNA were roughly 167% greater. Alewife eDNA presence was also restricted to fewer Restored sites and was not detected upstream of Daniels Dam. Concentrations of eDNA are known to be strongly correlated with relative fish abundance and biomass density as demonstrated through field and experimental studies of several anadromous species [53, 54], including river herring in the Chesapeake Bay [35]. Therefore, the greater presence and concentration of blueback herring eDNA may be linked to a higher abundance of blueback herring in the Patapsco River post-dam removal. Analysis of river herring eDNA surveys at a region-wide scale suggest that river systems on the western shore of the Chesapeake Bay (including the Patapsco River) typically have greater blueback herring presence than the eastern shore, and vice versa for alewife [50]. In multispecies studies of other migratory fish, species with greater abundances pre-removal also had the greatest increase in detection probability and upstream extent after a dam removal compared to rarer species [55]. There is also substantial interannual variability in river herring migration and spawning dynamics in the Chesapeake Bay [56]. Thus, the relatively greater response of blueback herring, based on our eDNA results, are likely river specific within the Chesapeake Bay or may have coincided with years when there were stronger blueback herring runs.

Distinct habitat preference and migration behaviors may also contribute to the observed differences in alewife and blueback herring eDNA data. Environmental factors such as water temperature, flow regime, and nutrient and chlorophyll concentration have been identified as potential drivers of variability in river herring run dynamics [56–58]. River discharge and substrate are also strong determinants spawning habitat suitability for each species [59]. Notably, alewife tend to spawn in slower moving streams and prefer gravel and pebbles for spawning, while blueback herring prefer faster flows over gravel, sand, and finer sediments [59, 60]. Dam removal can drastically alter the hydrology and stream bed morphology within streams as bank elevation and natural flow is restored [61]. In the Patapsco River, the removal of Bloede Dam is expected to shift the ecosystem as it reverts from lentic to a lotic state at the location of the former dam pond [34, 62].

### Advantages and limitations of early detection methods

By integrating all four methods to monitor migration and spawning activity across multiple reaches in the Patapsco River downstream and upstream of Bloede Dam, we present a robust analysis of the short-term response of river herring to dam removal. Each of the four monitoring techniques has its strengths and limitations. Ichthyoplankton sampling of egg and larval counts can provide direct evidence of active spawning [25]. Egg releases by an individual fish may also magnify its presence over an area many times larger than the space occupied by the animal itself. However, species-specific analyses of such data can be difficult and may further be confounded by accidental inclusion of other visually similar species, such as hickory shad. Electrofishing and PIT telemetry are widely used to assess the distribution of fish in relation to habitat features, including dams (electrofishing: [31, 63]; PIT tagging: [27, 41]). While PIT tags enable tracking of migration patterns for individual fish at a fine timescale, tag loss is a major constraint and detection data may not be reliable or accurate if, for instance, the tagged fish does not swim directly over the antenna or two overlapping tags pass the antenna simultaneously [64, 65]. There were also sampling challenges in the years after dam removal. High flow conditions and the COVID-19 pandemic made it difficult to deploy and maintain PIT antennas and conduct electrofishing during the 2019 and 2020 spring migration months. The antennas were removed during high flow events in 2019 and not deployed in the streambed for the entire 2020 sampling period.

Compared to other methods, electrofishing surveys have disadvantages as well as distinct advantages. The equipment is expensive, the surveys are labor intensive, and target species detections are generally more difficult than with other techniques. Whereas detection via eDNA or ichthyoplankton requires only that a water sample contain eggs or genetic material released by an individual fish within a detectible distance and time [68, 69], researchers conducting electrofishing surveys must navigate a boat within a few meters of the fish to incapacitate and capture it. Additional limitations include a low probability of capturing early migrants into restored habitats if they are present in low densities or with patchy distributions. Regardless of these issues, presence data from electrofishing surveys provide immediate, tangible, and certain identification of adult fish not available with other methods. Furthermore, regulators value these results, and detection of river herring using electrofishing surveys was one of the criteria for determining the success of the Bloede Dam removal for fish passage restoration [66]. Additionally, electrofishing can provide information on the size and sex composition of the spawning run via sample collection for determining fish age (otoliths), spawning history (scales), and population genetics (fin clips).

This study further demonstrates the practical application of eDNA as an early detection tool to document spatial patterns of anadromous fish habitat use in response to restoration. Our study adds to the growing body of literature using eDNA to assess aquatic habitat connectivity, spatial distribution, and restoration dynamics for anadromous fish populations [52, 54, 67]. We were more likely to detect eDNA in the river than ichthyoplankton, PIT tagged fish, or adult fish in electrofishing samples in the years immediately after the dam removal. These results indicate that changes in eDNA can be detected in aquatic streams before population-level abundance changes are detectable through traditional sampling methods. The eDNA assay used is highly sensitive, with consistent and robust amplification for river herring DNA [35]. Thus, positive eDNA detections indicate true species presence at or upstream of the sampling location. Detectable eDNA signals in freshwater systems can also typically last a few hundred meters downstream from the source and can persist from 1 to 54 days before degrading and dissipating [68, 69]. Therefore, positive detections of river herring eDNA are likely from local and recent sources of genetic material released during migration or spawning activity.

Despite the benefits of using eDNA as a monitoring tool, there are also potential limitations when interpreting eDNA results. Environmental factors can affect eDNA detectability and degradation, including a combination of hydrological conditions, UV exposure, and water temperature [53]. Detectability of eDNA may also depend on species density [52]. Thus, it is possible that some samples were false negatives, if the fish were indeed present at a location but in small enough numbers that the eDNA was too diffuse to detect. This may also explain why we observed reduced consistency in detecting river herring eDNA across duplicate samples moving upstream in the Patapsco River. Finally, eDNA data can face issues of overdispersion due to patchy spacing and density of fish in the environment (i.e., migration spikes) and movement of genetic material available to be sampled [70].

### Management implications

Removing dams is an effective strategy to reconnect habitats and restore anadromous fish populations, particularly alosines. Our study suggests that the primary objective for Bloede Dam’s removal—to restore migratory fish passage in the Patapsco River—has been met for river herring [71]. These trends of recovery are consistent with a region-wide assessment of river herring eDNA across the Chesapeake Bay, which detected river herring at the majority of sampling locations upstream of five former dam sites [50]. In a 2019 Endangered Species Act listing review for river herring, “the present or threatened destruction, modification, or curtailment of habitat or range” due to dams was ranked as the highest threat to alewife and blueback herring [18]. Over 400 dams remain standing in Maryland’s rivers as potential barriers to anadromous fish migration [72]. Notably, 28 dams were constructed pre-1950 and are now functionally obsolete, serving recreational purposes only [73]. Of these dams, ten were classified as “high” or “significant” hazard potential by the U.S. Army Corp of Engineers. As only 21 dams have been removed from rivers in Maryland since 1990, there are additional opportunities to expand river herring spawning habitat by restoring stream connectivity [74]. Dam removals fit into the broader strategy of “life cycle conservation,” which considers range-wide actions for key habitats critical for anadromous life cycles [75]. This approach has similarly been adopted in the recovery plans for other anadromous fishes, such as the endangered Coho salmon in California [76]. Furthermore, restoring aquatic connectivity for river herring spawning migrations can help recover additional ecological functions and ecosystem services along the ecosystem gradient of the watershed-ocean continuum [77]. River herring serve as forage prey for other birds, mammals, fish like the recreationally valuable striped bass, while transporting nutrients from the marine environment into freshwater ecosystems [78, 79]. Expanded ecological and biological monitoring of anadromous fishes pre-removal at priority dam sites can contribute valuable information to increase political, economic, and social support for this restoration strategy [61, 80].

Ultimately, the conservation end-goal for habitat and fish passage restoration is to improve stock productivity and recruitment. Nine out of the fifteen Chesapeake Bay stocks evaluated in the previous river herring stock assessment were either “overfished or severely depleted” [20]. The four survey methods presented in this study—eDNA, ichthyoplankton, electrofishing, and PIT tagging—only capture momentary and stationary snapshots of biological and ecological events. Additional assessment of river herring run counts in the Patapsco River, such as with imaging sonar, should be conducted to evaluate changes in breeding population size in response to the restoration effort [56]. Information on population-level responses can inform how spawning habitat restoration supplements other management actions, such as catch regulations that reduce harvest mortality and incidental by-catch [8, 24, 75]. Environmental monitoring of temperature, flow, and sediment condition in the river is also essential to characterizing habitat suitability post-restoration. Post-dam removal, elevated sediment loads and water column turbidity can extend to coastal subtidal zones and persist in the water column for years [81]. Thus, it is important to continue monitoring changes in the aquatic environment of the Patapsco River to understand how river herring and other fish species respond over time. Such additional considerations could be integrated into alternative analyses and environmental assessments for future dam removal projects [34].

As our results suggest, both alewife and blueback herring began using habitat upstream of the Bloede Dam site almost immediately after dam removal, but their responses were species-specific and context dependent. River herring are currently managed collectively under the Fishery Management Plan for American Shad and River Herring, which considers alewife and blueback herring together [21]. However, the species-specific responses observed in this study suggest that fish passage restoration and other conservation and management actions may benefit from species-specific analyses. In addition, different Chesapeake Bay rivers host different genetic stocks of alewife and blueback herring [82], which might also exhibit different responses to fish passage restoration. The integrated approach of this study provided rich context for understanding the early stages of restoration response. We documented rapid expansion of river herring into re-opened habitat while also showing that a small fraction of the population participated in the expansion to date and that spawning activity in the re-opened habitat, if it is happening, remains at undetectable levels. Similar approaches could be valuable for understanding short and longer-term responses of anadromous fish to fish passage restoration across a range of species and environmental contexts.

## Supporting information

S1 Fig Sampling effort for eDNA and ichthyoplankton in the Patapsco River. Samples were collected across 16 sites and seven years (2015 to 2021), and sites are arranged in order from farthest upstream to lowest downstream. Locations of each site are in S1 Fig.
(TIF)

S2 Fig Map of sampled reaches for Maryland Department of Natural Resources boat electrofishing in the Patapsco River from 2015 to 2021. Mapping layers (A) from Chesapeake Assessment and Scenario Tool (CAST) (2020), Maryland iMAP, Maryland Geological Survey, NOAA, Maryland Coastal Zone Management Program (2003), US Census Bureau (2018).
(TIF)

S3 Fig Map of PIT tag antenna locations in the Patapsco River from 2016 to 2021. “Total reach covered” refers to the total area of the river between individually deployed antennas that are considered as the same site for analysis purposes. Mapping layers (A) from Chesapeake Assessment and Scenario Tool (CAST) (2020), Maryland iMAP, Maryland Geological Survey, NOAA, Maryland Coastal Zone Management Program (2003), US Census Bureau (2018).
(TIF)

S4 Fig Male alewife and blueback herring collected in electrofishing surveys upstream of the former Bloede Dam site in 2021. Male alewife collected upstream of the former Bloede Dam site on March 26, 2021. Male blueback herring (B) collected in electrofishing samples upstream of the former Bloede Dam site on May 13, 2021. Images from Maryland Department of Natural Resources.
(TIF)

## References

[1] Pess GR, Quinn TP, Gephard SR, Saunders R. Re-colonization of Atlantic and Pacific rivers by anadromous fishes: linkages between life history and the benefits of barrier removal. Reviews in Fish Biology and Fisheries. 2014 Sep;24(3):881–900.

[2] Vannote RL, Minshall GW, Cummins KW, Sedell JR, Cushing CE. The river continuum concept. Canadian journal of fisheries and aquatic sciences. 1980 Jan 1;37(1):130–7.

[3] Dudgeon D, Arthington AH, Gessner MO, Kawabata ZI, Knowler DJ, Lévêque C, et al. Freshwater biodiversity: importance, threats, status and conservation challenges. Biological reviews. 2006 May;81(2):163–82. doi: 10.1017/S1464793105006950 16336747

[4] Liermann CR, Nilsson C, Robertson J, Ng RY. Implications of dam obstruction for global freshwater fish diversity. BioScience. 2012 Jun 1;62(6):539–48.

[5] Mattocks S, Hall CJ, Jordaan A. Damming, lost connectivity, and the historical role of anadromous fish in freshwater ecosystem dynamics. BioScience. 2017 Aug 1;67(8):713–28. 10.1093/biosci/bix069

[6] Hall CJ, Jordaan A, Frisk MG. Centuries of anadromous forage fish loss: consequences for ecosystem connectivity and productivity. BioScience. 2012 Aug 1;62(8):723–31. 10.1525/bio.2012.62.8.5

[7] Kemp PS, O’hanley JR. Procedures for evaluating and prioritising the removal of fish passage barriers: a synthesis. Fisheries Management and Ecology. 2010 Aug;17(4):297–322. 10.1111/j.1365-2400.2010.00751.x

[8] Hare JA, Borggaard DL, Alexander MA, Bailey MM, Bowden AA, Damon‐Randall K, et al. A Review of River Herring science in support of species conservation and ecosystem restoration. Marine and Coastal Fisheries. 2021 Dec;13(6):627–64.

[9] Noonan MJ, Grant JW, Jackson CD. A quantitative assessment of fish passage efficiency. Fish and Fisheries. 2012 Dec;13(4):450–64.

[10] Catalano MJ, Bozek MA, Pellett TD. Effects of dam removal on fish assemblage structure and spatial distributions in the Baraboo River, Wisconsin. North American Journal of Fisheries Management. 2007 May 1;27(2):519–30. 10.1577/M06-001.1

[11] Hogg R, Coghlan SM Jr, Zydlewski J. Anadromous sea lampreys recolonize a Maine coastal river tributary after dam removal. Transactions of the American Fisheries Society. 2013 Sep 1;142(5):1381–94. 10.1080/00028487.2013.811103

[12] Watson JM, Coghlan SM Jr, Zydlewski J, Hayes DB, Kiraly IA. Dam removal and fish passage improvement influence fish assemblages in the Penobscot River, Maine. Transactions of the American Fisheries Society. 2018 May;147(3):525–40.

[13] Duda JJ, Torgersen CE, Brenkman SJ, Peters RJ, Sutton KT, Connor HA, et al. Reconnecting the Elwha River: spatial patterns of fish response to dam removal. Frontiers in Ecology and Evolution. 2021:811. 10.3389/fevo.2021.765488

[14] Doyle MW, Harbor JM, Stanley EH. Toward policies and decision-making for dam removal. Environmental Management. 2003 Apr;31(4):0453–65. 10.1007/s00267-002-2819-z 12677292

[15] Wippelhauser G. Recovery of diadromous fishes: A Kennebec River case study. Transactions of the American Fisheries Society. 2021 May;150(3):277–90. 10.1002/tafs.10292.

[16] Atkins CG, Foster N. First Report of the Commissioners of Fisheries of the State of Maine, 1867. Owen and Nash, Printers to the State, Augusta, ME. 1868.

[17] Limburg KE, Waldman JR. Dramatic declines in North Atlantic diadromous fishes. BioScience. 2009 Dec 1;59(11):955–65. 10.1525/bio.2009.59.11.7

[18] NMFS. Endangered and threatened wildlife and plants; Endangered Species Act listing determination for Alewife and Blueback Herring. Federal Register 84:118. 19 June 2019:28630–28666.

[19] Fay CW, Neves RJ, Pardue GB. Species profiles: life histories and environmental requirements of coastal fishes and invertebrates (mid-Atlantic)—alewife/blueback herring. US Fish and Wildlife Service, Division of Biological Services, FWS/OBS-82111.9. US Army Corps of Engineers, TR EL-82-4; 1983;82(11).

[20] Atlantic States Marine Fisheries Commission (ASMFC). Amendment 2 to the Interstate Fishery Management Plan for Shad and River Herring. ASMFC, Arlington, Va. 2012. Available from: https://jcaa.org/News/amendment2_RiverHerring.pdf

[21] Atlantic States Marine Fisheries Commission (ASMFC). River Herring Stock Assessment Update Volume II: State‐Specific Reports. 2017. Available from: http://www.asmfc.org/uploads/file/59c2ac1fRiverHerringStockAssessmentUpdateVolumeII_State-Specific_Aug2017.pdf

[22] Palkovacs EP, Hasselman DJ, Argo EE, Gephard SR, Limburg KE, Post DM, et al. Combining genetic and demographic information to prioritize conservation efforts for anadromous alewife and blueback herring. Evolutionary Applications. 2014 Feb;7(2):212–26. doi: 10.1111/eva.12111 24567743 PMC3927884

[23] Davis JP, Schultz ET, Vokoun JC. Striped Bass consumption of Blueback Herring during vernal riverine migrations: does relaxing harvest restrictions on a predator help conserve a prey species of concern?. Marine and Coastal Fisheries. 2012 Jan 1;4(1):239–51.

[24] Dias BS, Frisk MG, Jordaan A. Contrasting fishing effort reduction and habitat connectivity as management strategies to promote alewife (Alosa pseudoharengus) recovery using an ecosystem model. Limnology and Oceanography. 2022 Feb;67:S5–22. 10.1080/19425120.2012.675972

[25] Burdick SM, Hightower JE. Distribution of spawning activity by anadromous fishes in an Atlantic slope drainage after removal of a low-head dam. Transactions of the American Fisheries Society. 2006 Sep 1;135(5):1290–300. 10.1577/T05-190.1

[26] Raabe JK, Hightower JE. Assessing distribution of migratory fishes and connectivity following complete and partial dam removals in a North Carolina river. North American Journal of Fisheries Management. 2014 Sep 3;34(5):955–69.

[27] Raabe JK, Hightower JE. American Shad migratory behavior, weight loss, survival, and abundance in a North Carolina river following dam removals. Transactions of the American Fisheries Society. 2014 May 4;143(3):673–88.

[28] Hogg RS, Coghlan SM Jr, Zydlewski J, Gardner C. Fish community response to a small-stream dam removal in a maine coastal river tributary. Transactions of the American Fisheries Society. 2015 May 4;144(3):467–79. 10.1080/00028487.2015.1007164

[29] The Chesapeake Bay Program. Chesapeake Bay Watershed Agreement. 2020. Available from: www.chesapeakebay.net/documents/FINAL_Ches_Bay_Watershed_Agreement.withsignatures-HIres.pdf.

[30] Roy SG, Uchida E, de Souza SP, Blachly B, Fox E, Gardner K, et al. A multiscale approach to balance trade-offs among dam infrastructure, river restoration, and cost. Proceedings of the National Academy of Sciences. 2018 Nov 20;115(47):12069–74. doi: 10.1073/pnas.1807437115 30397124 PMC6255187

[31] Harbold W., Kilian J., Graves P., & Mullaney M. Patapsco River Dam Removal Study: Assessing Changes in American Eel Distribution and Aquatic Communities, 2013–2014 Biennial Report. Prepared for: Maryland Environmental Service. 2015. Available from: https://dnr.maryland.gov/streams/Publications/2013-2014_PatapscoDamRemoval_BiologicalMonitoringReport.pdf

[32] O’Dell, J., J. Gabor, R. Dintaman. Survey of anadromous fish spawning areas. Completion Report, Project AFC-8 July 1970 –January 1975 for Potomac River Drainage and Upper Chesapeake Bay Drainage. Maryland Department of Natural Resources, Fisheries Administration, Anadromous Fish Survey Program. Annapolis, Maryland. 1975.

[33] Harbold W., Stranko S., Kilian J., Ashton M., Graves P., & McClain S. Patapsco River Dam removal study: Assessing changes in American Eel distribution and aquatic communities. MDNR, MNAD, Annapolis, MD. 2013. Available from: https://dnr.maryland.gov/streams/Publications/2012_PatapscoFinalReport.pdf

[34] Pess GR, McHenry ML, Beechie TJ, Davies J. Biological impacts of the Elwha River dams and potential salmonid responses to dam removal. Northwest Science. 2008 Dec;82(sp1):72–90.

[35] Melchior M., Norris W., Lowe S., Boardman G., Ditchey E. Bloede Dam Alternatives Analysis, prepared by Interfluve for American Rivers. 2012. Available from: https://dnr.maryland.gov/fisheries/Documents/BloedeAltAnalysisFinal_061412.pdf

[36] Renshaw MA, Olds BP, Jerde CL, McVeigh MM, Lodge DM. The room temperature preservation of filtered environmental DNA samples and assimilation into a phenol–chloroform–isoamyl alcohol DNA extraction. Molecular ecology resources. 2015 Jan;15(1):168–76. doi: 10.1111/1755-0998.12281 24834966 PMC4312482

[37] Carr IM, Robinson JI, Dimitriou R, Markham AF, Morgan AW, Bonthron DT. Inferring relative proportions of DNA variants from sequencing electropherograms. Bioinformatics. 2009 Dec 15;25(24):3244–50. 10.1093/bioinformatics/btp583 19819885

[38] Uphoff J., McGinty M., Lukacovic R., Mowrer J., & Pyle B. Marine and estuarine finfish ecological and habitat investigations. Maryland Department of Natural Resources Report. 2017. Available from: https://dnr.maryland.gov/fisheries/Documents/F%2063%20R%208%20final.pdf

[39] Keller AA, Klein-MacPhee G, Burns JS. Abundance and distribution of ichthyoplankton in Narragansett Bay, Rhode Island, 1989–1990. Estuaries. 1999 Mar;22(1):149–63.

[40] Loesch JG, Lund WA JR. A contribution to the life history of the blueback herring, Alosa aestivalis. Transactions of the American Fisheries Society. 1977 Nov;106(6):583–9.

[41] Castro-Santos T, Haro A, Walk S. A passive integrated transponder (PIT) tag system for monitoring fishways. Fisheries research. 1996 Oct 1;28(3):253–61.

[42] Castro-Santos T, Vono V. Posthandling survival and PIT tag retention by Alewives—a comparison of gastric and surgical implants. North American Journal of Fisheries Management. 2013 Aug 1;33(4):790–4. 10.1080/02755947.2013.811130

[43] Brooks ME, Kristensen K, Van Benthem KJ, Magnusson A, Berg CW, Nielsen A, et al. glmmTMB balances speed and flexibility among packages for zero-inflated generalized linear mixed modeling. The R journal. 2017;9(2):378–400.

[44] R Core Development Team. R: a language and environment for statistical computing [Internet]. Vienna, Austria: R Foundation for Statistical Computing; 2021. https://www.R-project.org

[45] Mullahy J. 1986. Specification and testing of some modified count data models. Journal of econometrics, 33: 341–365.

[46] Searle SR, Speed FM, Milliken GA. Population marginal means in the linear model: an alternative to least squares means. The American Statistician. 1980 Nov 1;34(4):216–21.

[47] Lenth R., Singmann H., Love J., Buerkner P., & Herve M. Package ‘emmeans’. R package version 0.2.0. 2019. Available from: https://cran.r-project.org/web/packages/emmeans/

[48] Hartig F. DHARMa: residual diagnostics for hierarchical (multi-level/mixed) regression models. R package version 0.2.0. 2018. Available from: http://florianhartig.github.io/DHARMa/

[49] Jessop BM. Homing of alewives (Alosa pseudoharengus) and blueback herring (A. aestivalis) to and within the Saint John River, New Brunswick, as indicated by tagging data. Department of Fisheries and Oceans, Biological Sciences Branch, Freshwater and Anadromous Division; 1994.

[50] Ogburn MB, Plough LV, Bangley CW, Fitzgerald CL, Hannam MP, Lee B, et al. Environmental DNA reveals anadromous river herring habitat use and recolonization after restoration of aquatic connectivity. Environmental DNA. 2022. 10.1002/edn3.348

[51] Thomsen PF, Willerslev E. Environmental DNA–An emerging tool in conservation for monitoring past and present biodiversity. Biological conservation. 2015 Mar 1;183:4–18.

[52] Muha TP, Rodriguez-Barreto D, O’Rorke R, Garcia de Leaniz C, Consuegra S. Using eDNA metabarcoding to monitor changes in fish community composition after barrier removal. Frontiers in Ecology and Evolution. 2021:28.

[53] Tillotson MD, Kelly RP, Duda JJ, Hoy M, Kralj J, Quinn TP. Concentrations of environmental DNA (eDNA) reflect spawning salmon abundance at fine spatial and temporal scales. Biological Conservation. 2018 Apr 1;220:1–1.

[54] Spear MJ, Embke HS, Krysan PJ, Vander Zanden MJ. Application of eDNA as a tool for assessing fish population abundance. Environmental DNA. 2021 Jan;3(1):83–91. 10.1002/edn3.94

[55] Duda JJ, Hoy MS, Chase DM, Pess GR, Brenkman SJ, McHenry MM, et al. Environmental DNA is an effective tool to track recolonizing migratory fish following large‐scale dam removal. Environmental DNA. 2021 Jan;3(1):121–41. 10.1002/edn3.134

[56] Ogburn MB, Spires J, Aguilar R, Goodison MR, Heggie K, Kinnebrew E, et al. Assessment of river herring spawning runs in a Chesapeake Bay coastal plain stream using imaging sonar. Transactions of the American Fisheries Society. 2017 Jan 2;146(1):22–35.

[57] Legett HD, Jordaan A, Roy AH, Sheppard JJ, Somos‐Valenzuela M, Staudinger MD. Daily patterns of river herring (Alosa spp.) spawning migrations: environmental drivers and variation among coastal streams in Massachusetts. Transactions of the American Fisheries Society. 2021 Jul;150(4):501–13.

[58] Bi R, Jiao Y, Weaver LA, Greenlee B, McClair G, Kipp J, et al. Environmental and anthropogenic influences on spatiotemporal dynamics of Alosa in Chesapeake Bay tributaries. Ecosphere. 2021 Jun;12(6):e03544.

[59] Greene KE, Zimmerman JL, Laney RW, Thomas-Blate JC. Atlantic coast diadromous fish habitat: a review of utilization, threats, recommendations for conservation, and research needs. Atlantic States Marine Fisheries Commission Habitat Management Series. 2009 Jan;464:276.

[60] Loesch JG. Overview of life history aspects of anadromous alewife and blueback herring in freshwater habitats. InAmerican Fisheries Society Symposium 1987 (Vol. 1).

[61] Bednarek AT. Undamming rivers: a review of the ecological impacts of dam removal. Environmental management. 2001 Jun;27(6):803–14. 10.1007/s002670010189 11393315

[62] Harbold W., Watson J., Kilian J., & Stranko S., Biological Monitoring and American Eel Passage in the Patapsco River, Maryland from 2013–2017. MDNR, MNAD, Annapolis, MD. 2018.

[63] Bain MB, Finn JT, Booke HE. A quantitative method for sampling riverine microhabitats by electrofishing. North American Journal of Fisheries Management. 1985 Jul;5(3B):489–93. doi: 10.1577/1548-8659(1985)5<489:AQMFSR>2.0.CO;2

[64] Gibbons WJ, Andrews KM. PIT tagging: simple technology at its best. Bioscience. 2004 May 1;54(5):447–54.

[65] Saboret G, Dermond P, Brodersen J. Using PIT‐tags and portable antennas for quantification of fish movement and survival in streams under different environmental conditions. Journal of Fish Biology. 2021 Aug;99(2):581–95. doi: 10.1111/jfb.14747 33821479

[66] Maryland State Highway Administration. Bloede Dam Mitigation Bank Prospectus. 2017. Available from: http://www.nab.usace.army.mil/Missions/Regulatory/Public-Notices/

[67] Pfleger MO, Rider SJ, Johnston CE, Janosik AM. Saving the doomed: Using eDNA to aid in detection of rare sturgeon for conservation (Acipenseridae). Global Ecology and Conservation. 2016 Oct 1;8:99–107.

[68] Barnes MA, Turner CR. The ecology of environmental DNA and implications for conservation genetics. Conservation genetics. 2016 Feb;17(1):1–7.

[69] Wilcox TM, McKelvey KS, Young MK, Sepulveda AJ, Shepard BB, Jane SF, et al. Understanding environmental DNA detection probabilities: A case study using a stream-dwelling char Salvelinus fontinalis. Biological Conservation. 2016 Feb 1;194:209–16. doi: 10.1016/j.biocon.2015.12.023

[70] Chambert T, Pilliod DS, Goldberg CS, Doi H, Takahara T. An analytical framework for estimating aquatic species density from environmental DNA. Ecology and evolution. 2018 Mar;8(6):3468–77. 10.1002/ece3.3764 29607039 PMC5869225

[71] American Rivers. Removing Bloede: American Rivers’ Quest to Free the Patapsco River. 2018. Available from: https://www.americanrivers.org/patapsco/index.html.

[72] Maryland Department of the Environment. Maryland Dam Inventory. 2021. Available from: https://mde.maryland.gov/programs/Water/DamSafety/Pages/maryland_dam_inventory.aspx

[73] US Army Corps of Engineers. National Dam Inventory. 2021.

[74] American Rivers. Database of Dam Removals in the U.S. Raw Dataset—ARDamRemovalList_figshare_Feb2022. 2022. Figshare. Available from: 10.6084/m9.figshare.5234068

[75] Bowden AA. Towards a comprehensive strategy to recover river herring on the Atlantic seaboard: lessons from Pacific salmon. ICES Journal of Marine Science. 2014 Mar;71(3):666–71.

[76] NMFS (National Marine Fisheries Service), Final Recovery Plan for Central California Coast Coho salmon (Oncorhynchus kisutch) Evolutionarily Significant Unit, Santa Rosa, CA, National Marine Fisheries Service Southwest Region. 2012

[77] Ouellet V, Collins MJ, Kocik JF, Saunders R, Sheehan TF, Ogburn MB, et al. The diadromous watersheds-ocean continuum: Managing diadromous fish as a community for ecosystem resilience. Frontiers in Ecology and Evolution. 2022 Nov 08;10:1–29.

[78] MacAvoy SE, Macko SA, McIninch SP, Garman GC. Marine nutrient contributions to freshwater apex predators. Oecologia. 2000 Mar;122(4):568–73. doi: 10.1007/s004420050980 28308350

[79] West DC, Walters AW, Gephard S, Post DM. Nutrient loading by anadromous alewife (Alosa pseudoharengus): contemporary patterns and predictions for restoration efforts. Canadian Journal of Fisheries and Aquatic Sciences. 2010 Aug;67(8):1211–20.

[80] Rodeles AA, Galicia D, Miranda R. Recommendations for monitoring freshwater fishes in river restoration plans: a wasted opportunity for assessing impact. Aquatic Conservation: Marine and Freshwater Ecosystems. 2017 Aug;27(4):880–5.

[81] Rubin SP, Miller IM, Foley MM, Berry HD, Duda JJ, Hudson B, et al. Increased sediment load during a large-scale dam removal changes nearshore subtidal communities. PloS one. 2017 Dec 8;12(12):e0187742. doi: 10.1371/journal.pone.0187742 29220368 PMC5722376

[82] Ogburn MB, Hasselman DJ, Schultz TF, Palkovacs EP. Genetics and juvenile abundance dynamics show congruent patterns of population structure for depleted river herring populations in the upper Chesapeake Bay. North American Journal of Fisheries Management. 2017b Sep 3;37(5):1083–92.

