# Supplementary figures and images for: Early detection and recovery of river herring spawning habitat use in response to a mainstem dam removal
Source: PLoS One. 2023 May 3;18(5):e0284561. doi: 10.1371/journal.pone.0284561 (PMC10156059; doi:10.1371/journal.pone.0284561)

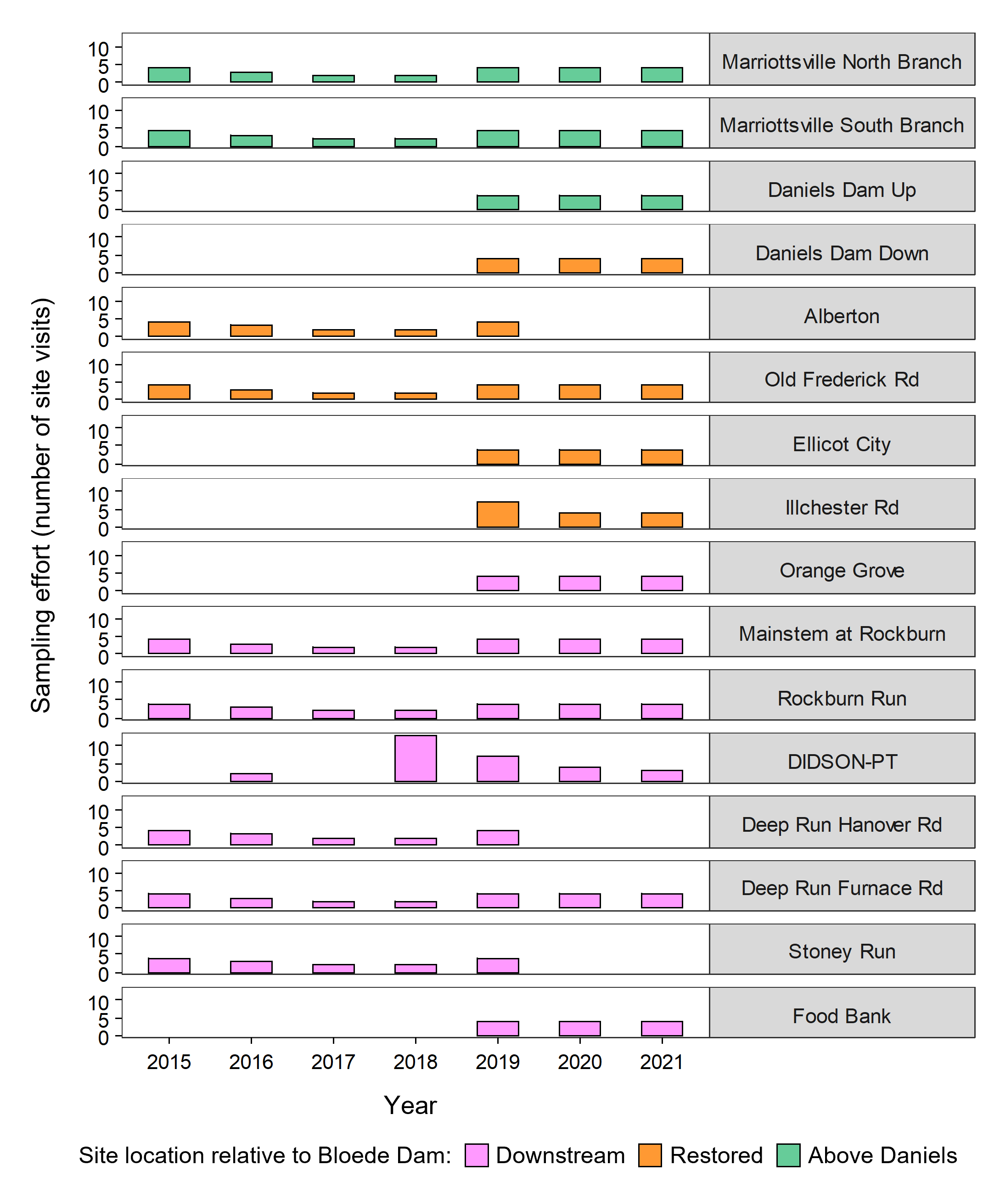

Supplement: S1 Fig — Samples were collected across 16 sites and seven years (2015 to 2021), and sites are arranged in order from farthest upstream to lowest downstream. Locations of each site are in S1 Fig. (TIF) [file pone.0284561.s001.tif]

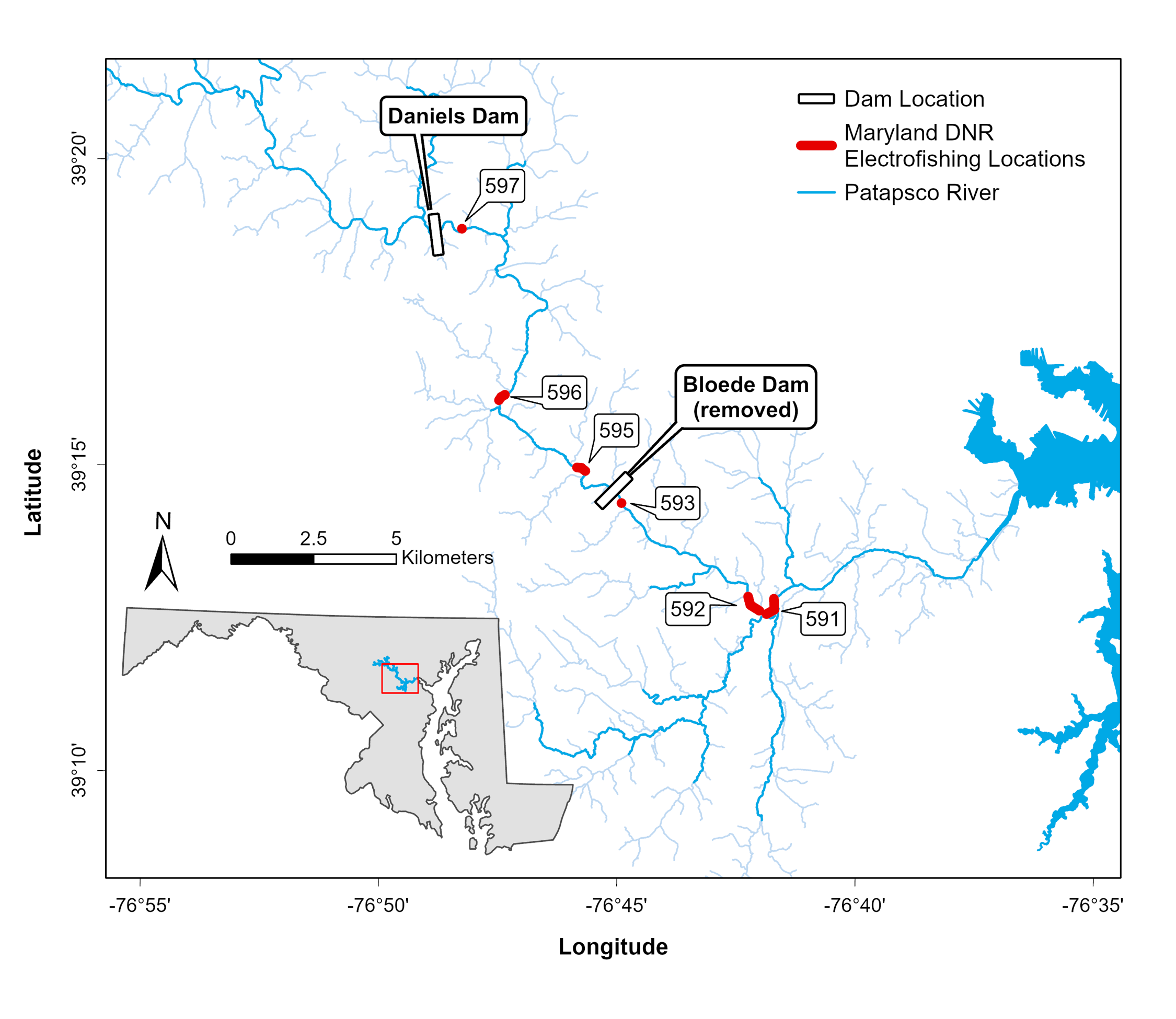

Supplement: S2 Fig — Mapping layers (A) from Chesapeake Assessment and Scenario Tool (CAST) (2020), Maryland iMAP, Maryland Geological Survey, NOAA, Maryland Coastal Zone Management Program (2003), US Census Bureau (2018). (TIF) [file pone.0284561.s002.tif]

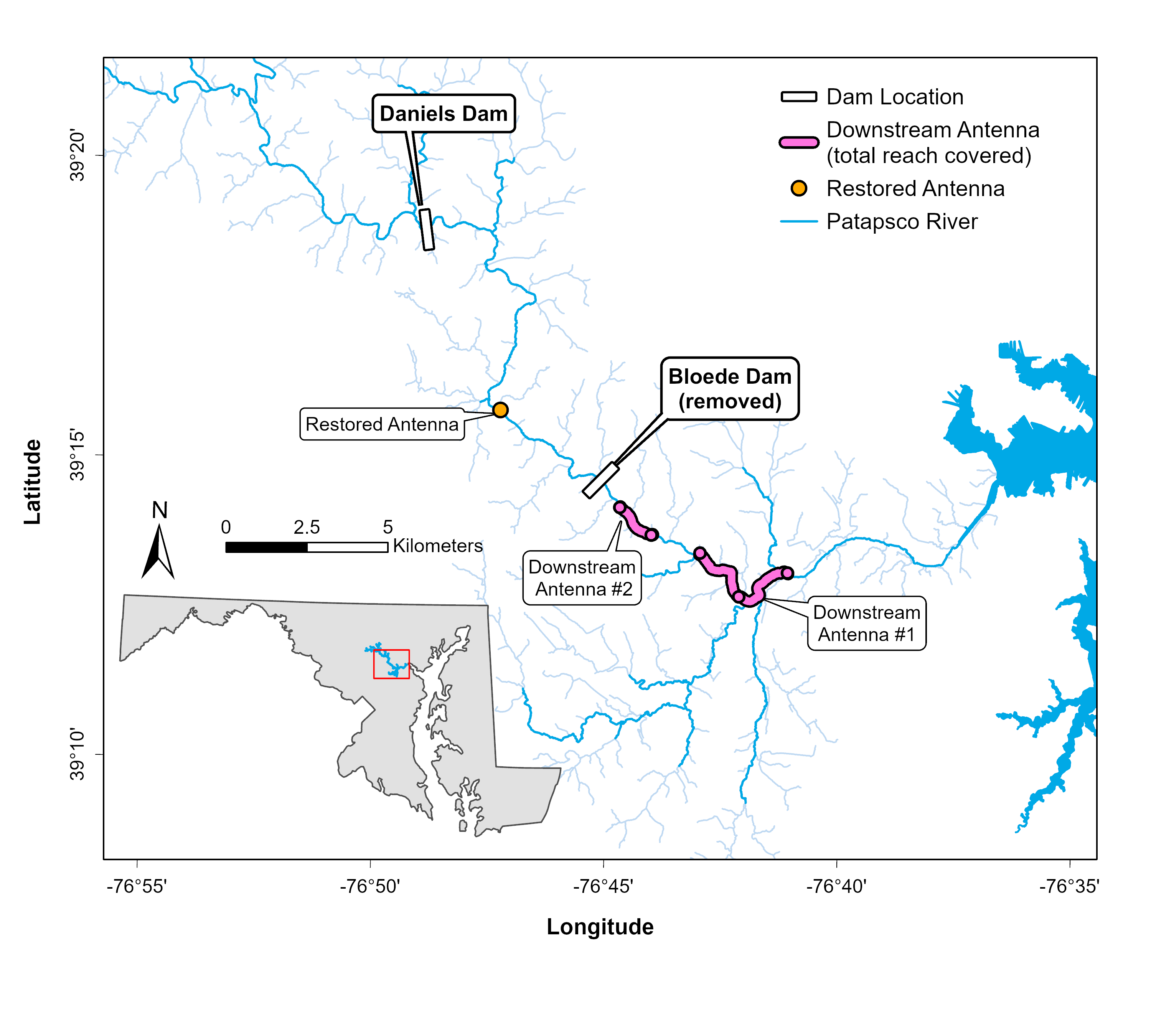

Supplement: S3 Fig — “Total reach covered” refers to the total area of the river between individually deployed antennas that are considered as the same site for analysis purposes. Mapping layers (A) from Chesapeake Assessment and Scenario Tool (CAST) (2020), Maryland iMAP, Maryland Geological Survey, NOAA, Maryland Coastal Zone Management Program (2003), US Census Bureau (2018). (TIF) [file pone.0284561.s003.tif]

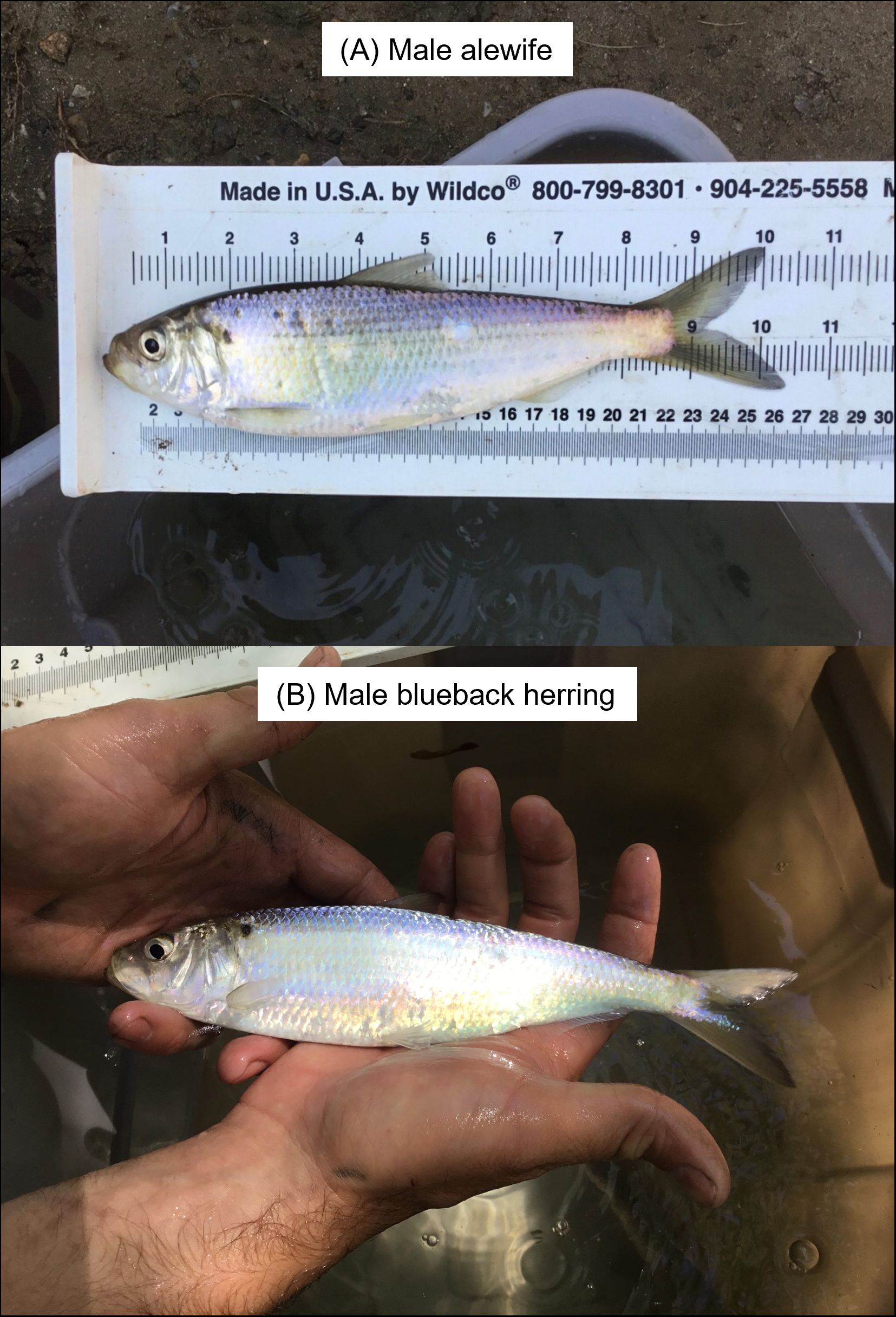

Supplement: S4 Fig — Male alewife collected upstream of the former Bloede Dam site on March 26, 2021. Male blueback herring (B) collected in electrofishing samples upstream of the former Bloede Dam site on May 13, 2021. Images from Maryland Department of Natural Resources. (TIF) [file pone.0284561.s004.tif]
